# Unsupervised phenotypic clustering, inferential characterization, and discriminant classification of elite flatwater canoeists: neuromuscular and anthropometric determinants

**DOI:** 10.3389/fphys.2026.1949031

**Published:** 2026-10-19

**Authors:** Tania Álvarez-Yates, Óscar García-García, Alba Cuba-Dorado, José-Benito Bouza-Rodríguez, Alberto Comesaña-Campos, Jorge Cerqueiro-Pequeño

**Affiliations:** 1 Department of Physical Education and Sports, Faculty of Sports Sciences and Physical Education, University of A Coruña, A Coruña, Spain; 2 Sport Performance, Physical Condition and Wellness lab, Faculty of Education and Sports Sciences, University of Vigo, Pontevedra, Spain; 3 Sports Research Institute (CIDEGA), Faculty of Education and Sport Sciences, University of Vigo, Vigo, Spain; 4 Department of Design in Engineering, School of Industrial Engineering, University of Vigo, Vigo, Spain

**Keywords:** elite canoeists, flatwater sprint canoeing, phenotypic profiling, sexual dimorphism, unsupervised clustering

## Abstract

**Introduction:**

Phenotypic profiling of elite athletes remains constrained by supervised frameworks that conflate physiological characteristics with performance outcomes. Unsupervised machine learning offers an outcome-independent alternative for identifying intrinsic athlete profiles.

**Method:**

This study applied clustering algorithms to comprehensive laboratory data from elite flatwater canoeists. Twenty-one international-level C1 canoeists (12 male, 9 female) underwent a standardized testing battery including body composition, muscle contractile properties, mobility, maximal and explosive strength, and sport-specific neuromuscular assessments via Functional Electromechanical Dynamometry (FEMD). All performance variables were excluded to ensure purely phenotypic segmentation. Preprocessing included asymmetric winsorization, Yeo-Johnson transformation, and robust scaling. K-Means and hierarchical clustering (Ward linkage) were applied with optimal k determined via silhouette coefficient, Davies-Bouldin index, and elbow method. Discriminant variable importance was assessed via Random Forest Classifier; interpretable classification rules were extracted using Decision Tree analysis (max_depth=3) with stratified train-test validation (66.7%–33.3%). Statistical characterization of cluster differences employed ANOVA or Kruskal-Wallis tests with *post-hoc* comparisons and Benjamini-Hochberg FDR correction; algorithmic concordance was quantified via Adjusted Rand Index (ARI).

**Results:**

Three phenotypic clusters emerged: High-Neuromuscular Male Phenotype (n=7, 33.3%), Female-Dominant Phenotype (n=10, 47.6%), and Moderate-Neuromuscular Male Phenotype (n=4, 19.0%) (ARI = 0.591). Sex stratification was marked (χ²=18.90, p<0.001): the Female-Dominant Phenotype comprised 90% female athletes; both male phenotypes were exclusively male, encoding sexual dimorphism alongside intra-sex neuromuscular differentiation. FEMD neuromuscular variables dominated discrimination (75.9% of top-15 importance; 14 of 15 variables with $p_{\text{adj}}$ < 0.01 following Benjamini-Hochberg FDR correction). Decision Tree classification achieved 100% training accuracy and 71.4% test accuracy (5/7; 95% CI: 29.0%–96.3%; p=0.045 vs. chance), though the small test partition constrains generalizability. Within male phenotypes, Clusters 0 and 2 demonstrated equivalent neuromuscular profiles (all p > 0.05), with phenotypic separation captured by latissimus dorsi sustain time from the non-paddle side; differences relative to the Female-Dominant Phenotype primarily reflect sexual dimorphism.

**Discussion:**

Unsupervised clustering suggests a tripartite phenotypic structure in elite canoeists consistent with sexual dimorphism and intra-sex neuromuscular differentiation, with FEMD-derived eccentric force as primary discriminant. Classification rules may inform future sex-stratified talent identification and training individualization; external validation with larger cohorts is required.

## 1 Introduction

### 1.1 Athlete profiling and performance determinants in high-performance sport

Long-term athletic development models emphasize the importance of individualized training prescription based on athlete-specific characteristics (Johnston et al., 2018; Leite et al., 2021; Pichardo et al., 2018). In high-performance contexts, understanding sport-specific factors that influence competitive success requires detailed analysis of physical, physiological, and neuromuscular determinants (McGuigan, 2017). Phenotypic characterization and athlete profiling have become fundamental tools to optimize performance development, reduce preparation uncertainty, and support evidence-based talent identification (Bourdon et al., 2017; McGuigan, 2017).

In flatwater sprint canoeing, success depends on complex interactions between biomechanical, anthropometric, and neuromuscular variables, whose relative importance varies by race distance and boat type (Álvarez-Yates and García-García, 2021). Previous studies have highlighted the relevance of upper-body maximum strength and power (Kristiansen et al., 2023; Nilsson and Rosdahl, 2016; Van Someren and Howatson, 2008; Van Someren and Palmer, 2003), aerobic and anaerobic capacity (Bishop, 2000; Van Someren and Howatson, 2008; Van Someren and Palmer, 2003), and body composition (López-Plaza et al., 2017; Van Someren and Howatson, 2008; Van Someren and Palmer, 2003). However, discrepancies regarding which variables are most determinant indicate that current models lack the specificity and robustness necessary for widespread application.

### 1.2 Functional electromechanical dynamometry in canoe-specific assessment

Recent research has evolved toward approaches incorporating neuromuscular and biomechanical information specific to paddle stroke technique (Álvarez-Yates et al., 2024a, Álvarez-Yates et al., 2024b). Functional Electromechanical Dynamometry (FEMD) has emerged as a valid and reliable tool for assessing strength in sport-specific multi-joint patterns, enabling test design in positions analogous to canoe technique (Chamorro et al., 2018; Dvir and Müller, 2020; Rodriguez-Perea et al., 2019; Vega et al., 2018). Studies in high-level canoeists have demonstrated moderate-to-strong associations between FEMD-derived variables (maximum strength, fatigue resistance in canoe-specific position) and C1–500 m performance time (Álvarez-Yates et al., 2026). Sport-specific neuromuscular variables, such as stroke repetitions to failure in incremental tests, explain considerable competitive performance variance, reinforcing the importance of highly specific assessments (Álvarez-Yates et al., 2026).

### 1.3 Unsupervised machine learning for phenotypic segmentation in sport

Beyond outcome-focused performance modelling, unsupervised machine learning techniques have expanded possibilities for athlete profiling (Alvero-Cruz et al., 2020; Lerebourg et al., 2022; Vickers and Vertosick, 2016). Combining dimensionality reduction (Principal Component Analysis [PCA]) with clustering methods (K-means, hierarchical clustering) allows identification of athlete phenotypic profiles characterized by specific anthropometric, physiological, and neuromuscular patterns (Blythe and Király, 2016; Bunker and Susnjak, 2022; Claudino et al., 2019).

These procedures have successfully discriminated future achievement levels, defined athlete types, and supported decision-making in development programs. For example, Till et al. (2016) and Till and Baker (2020) applied cluster analysis to rugby players, identifying distinct physical profiles that predicted elite status. Despite established utility in team sports, no study has systematically applied machine learning-based phenotypic segmentation to elite canoeists using a comprehensive sport-specific neuromuscular assessment based on FEMD.

This gap is critical because: (a) individual variation in canoe-specific neuromuscular capacity may follow discrete phenotypic profiles rather than a single continuum; (b) bilateral force asymmetries due to unilateral paddle technique remain unexplored through clustering approaches; (c) the relative importance of different variable domains (anthropometric, neuromuscular, muscle contractile properties, body composition) in defining athlete phenotypes is unknown; and (d) talent identification frameworks lack empirically-derived athlete taxonomies for profile-specific interventions.

### 1.4 Objectives and hypotheses

Given the absence of unsupervised phenotypic segmentation in elite canoeists using FEMD-based assessment, this study aims to identify and characterize distinct phenotypic profiles in international-level flatwater canoeists using machine learning clustering techniques with explicit exclusion of performance outcome variables to ensure purely phenotypic classification. The specific objective is to segment elite canoeists into homogeneous clusters based on anthropometric, neuromuscular (FEMD-derived), muscle contractile properties and body composition, employing dimensionality reduction (PCA, t-distributed Stochastic Neighbor Embedding [t-SNE]) and clustering algorithms (K-Means, hierarchical Ward’s method), validated through internal consistency metrics (Silhouette coefficient, Davies-Bouldin index, Adjusted Rand Index).

The study will:

1. Determine optimal cluster number via convergence of multiple validation metrics.
2. Validate cluster stability through algorithmic concordance (K-Means vs. Ward, quantified via Adjusted Rand Index).
3. Characterize cluster phenotypes through inferential statistics (ANOVA/Kruskal-Wallis with *post-hoc* comparisons).
4. Identify discriminant variables using Random Forest Classifier feature importance.
5. Generate interpretable classification rules via Decision Tree analysis (*max_depth=3*) with stratified train-test validation.
6. Visualize cluster profiles via normalized radar charts with athlete rosters and descriptive statistics.

It is hypothesized that unsupervised clustering will reveal at least three phenotypically discernible data-driven patterns differentiated primarily by:

Neuromuscular variables from the sport-specific FEMD incremental test are expected to account for >50% of discriminant power, supporting FEMD as a primary tool for phenotypic profiling. Decision Tree classification rules are expected to achieve acceptable accuracy on independent test data, supporting potential prospective applicability. This data-driven taxonomy may inform individualized training prescription, asymmetry correction protocols, and talent identification frameworks tailored to distinct athlete profiles.

## 2 Material and methods

### 2.1 Study design and participants

A cross-sectional observational analysis was conducted on laboratory assessment data obtained from 21 elite flatwater canoeists competing at international level (C1 category, minimum national team representation). To preserve confidentiality, participants were assigned alphanumeric identifiers (S001–S021), with demographic stratification by sex presented in the Results section. Given the mixed-sex composition of the sample (12 males, 9 females) and the known magnitude of sexual dimorphism in neuromuscular and anthropometric variables, sex was treated as a *post-hoc* interpretive variable for cluster characterization rather than as a stratification criterion for the unsupervised segmentation phase. The resulting cluster structure is therefore expected to encode both sex-related and training-related phenotypic variance, and cluster labels are interpreted accordingly. All data were collected during routine laboratory assessments following approval from the local Ethics Research Committee (protocol code: 07-1019), with prior athlete consent in accordance with EU General Data Protection Regulation (GDPR 2016/679).

The primary methodological objective was to identify phenotypic profiles through unsupervised machine learning techniques applied to anthropometric, neuromuscular (FEMD-derived), muscle contractile properties and body composition variables. To ensure purely phenotypic segmentation without contamination from outcome measures, all performance-related variables, including race times over 1000, 500 and 200 m, ranking, and competition position, were explicitly excluded from the clustering process.

### 2.2 Data sources and assessment protocol

The primary data source consisted of a structured *.csv database containing 21 cases and 286 variables organized across four thematic domains. Demographic and anthropometric data included sex, country of origin, dominant paddle side, age, height, body weight, and body composition parameters assessed via bioelectrical impedance analysis (Tanita BC-601 Segment, Tanita Corporation, Tokyo, Japan) (Moon, 2013; L. C. Ward, 2019). Muscle contractile properties of the main muscles involved in canoe-stroke were assessed through tensiomyography (TMG). Maximal strength comprised 1-repetition maximum (1RM) values for bench press and bench pull exercises (Crisafulli et al., 2025), expressed both in absolute terms (kg) and normalized to body weight. Upper- and lower-limb mobility were assessed unilaterally using standardized tests. Upper limb mobility included shoulder range of motion (flexion and external/internal rotation) and dynamic stabilization evaluated through the Upper Quarter Y-Balance Test performance (anterior, posteromedial, and posterolateral reach with asymmetry indices) (Plisky et al., 2009), with reach distances normalized to upper-limb length measured as the C7-to-fingertips distance. Lower limb was assessed through the Active Knee Extension test for hamstring flexibility values (Davis et al., 2008). Explosive strength integrated a single-arm cable row performed in a canoe-specific position and a unilateral leg press, including asymmetry quantification (Álvarez-Yates et al., 2024a, Álvarez-Yates et al., 2024b). Specific canoe FEMD assessment included a maximal isometric force production in canoe-specific positioning, and an incremental load test to voluntary failure that captured repetitions completed, time under tension (concentric/eccentric/global phases), peak and mean force-velocity-power profiles, mechanical work, and impulse, all recorded in both absolute and body-weight-normalized formats. Laboratory assessment procedures adhered to the standardized protocol described by Álvarez-Yates et al (Álvarez-Yates et al., 2024a, Álvarez-Yates et al., 2024b; Álvarez-Yates et al., 2026).

The operational nomenclature of TMG and mobility variables are detailed in **Supplementary Table 1**, and the full set of explosive strength and FEMD-derived variables from isometric and incremental tests are summarized in **Supplementary Table 2**.

### 2.3 Data preprocessing pipeline

**Figure 1** illustrates the complete four-phase analytical workflow, with Phase 1 (Data Foundation & Preprocessing) detailed in this section. A sequential five-stage preprocessing pipeline was applied to ensure data quality and methodological rigor. First, performance-related variables (racing times, ranking, position) were excluded *a priori* from the analytical dataset prior to clustering, thereby guaranteeing unsupervised phenotypic segmentation without leakage of outcome information. The original source database comprised 286 variables per athlete. Of these, five were excluded prior to the analytical pipeline: three performance-outcome variables (t_1000m_, _500m_, t_200m_) and two competition-classification fields (category, medal standing), both of which encode competitive outcome information analogous to the performance variables and were therefore excluded under the same *a priori* rationale. The resulting analytical matrix comprised 281 numeric variables, which constitutes the phenotypic feature space referenced throughout Sections 3.2.1–3.2.5. Categorical identifiers (Athlete ID, Sex, Country, Paddle Side, Event Type) were similarly excluded from the numeric variable matrix.

**Figure 1:**
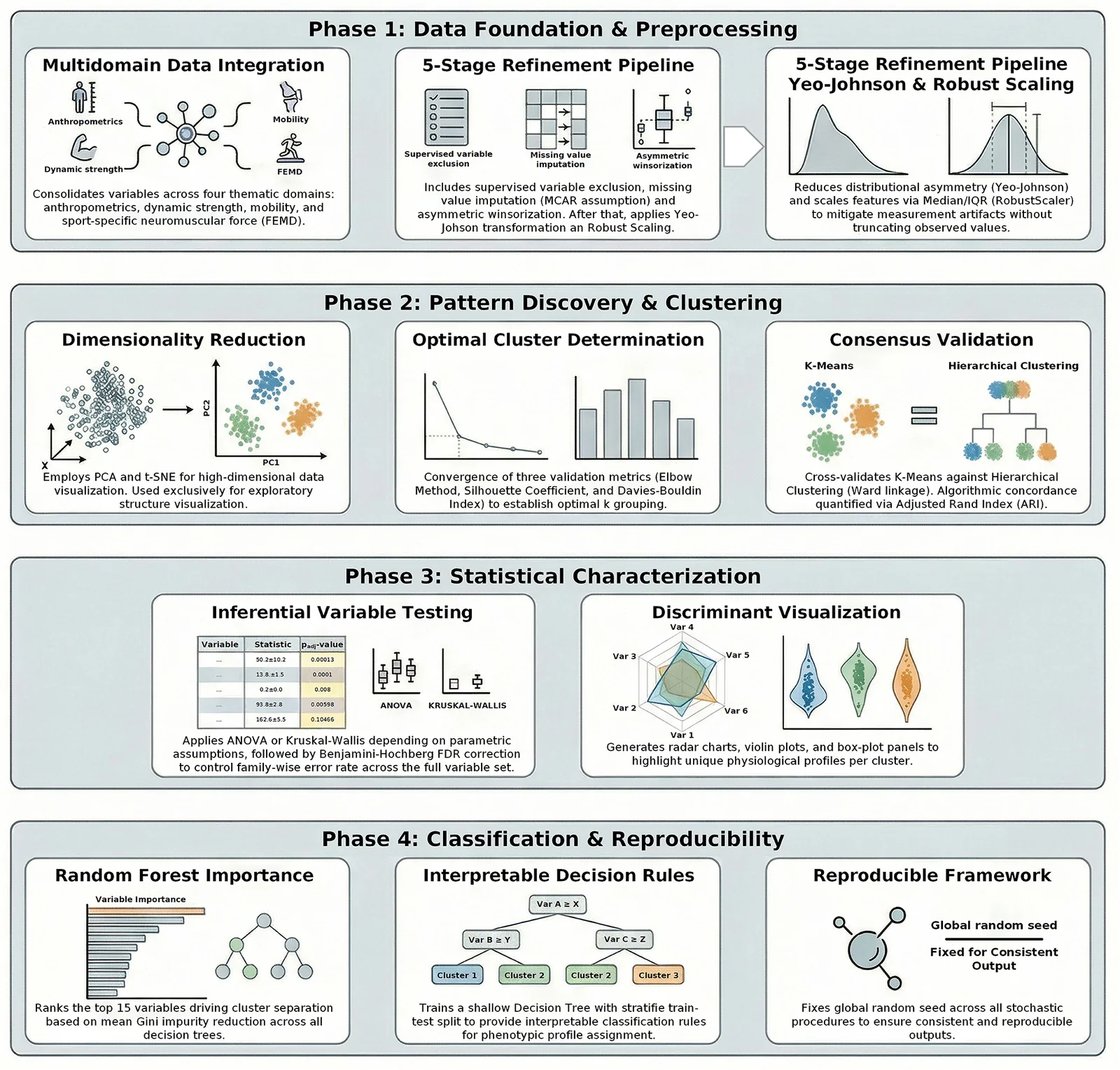
Analytical workflow for phenotypic clustering of elite canoeists. Four sequential phases: (1) Data Preprocessing: 286-variable integration with five-stage refinement (exclusion, imputation, winsorization, Yeo-Johnson transformation, robust scaling); (2) Pattern Discovery: PCA/t-SNE reduction, optimal k=3 determination via convergent metrics, K-Means/Hierarchical consensus (ARI = 0.591); (3) Statistical Characterization: ANOVA/Kruskal-Wallis testing with Benjamini-Hochberg FDR correction, sex stratification radar/violin visualizations; (4) Classification: Random Forest importance (top 15), Decision Tree rules (*max_depth=3*, 71.4% test accuracy, 95% CI via Clopper-Pearson exact binomial; p=0.045 vs. chance), reproducible seed=42 framework.

Second, no missing values were observed among the 21 valid athlete records for any of the 281 numeric variables used in clustering (missingness = 0%); consequently, no imputation was required for the analytic sample. A mean-imputation step (Little and Rubin, 2014) was retained in the preprocessing pipeline as a safeguard against missingness in future data extensions, but did not alter any observation in the present dataset. Third, asymmetric winsorization (Dixon, 1953) was implemented as a conservative outlier mitigation strategy. To preserve the full sample size (n=21) without case deletion, values below the 10th percentile were replaced with the 10th percentile value, while values exceeding the 95th percentile were capped at the 95th percentile​. This asymmetric approach was intentionally designed to protect elite performers (top 5%) while mitigating potential technical or measurement artifacts in extreme values. Descriptive statistics presented in Section 3.1 reflect raw, unprocessed values prior to this pipeline; minor discrepancies with cluster-level means in Section 3.2, specifically in age (20.0 vs. 19.5 years weighted), are attributable to Winsorization of one age outlier. Fourth, a Yeo-Johnson power transformation (Yeo and Johnson, 2000) was applied on a variable-by-variable basis using scikit-learn’s PowerTransformer (standardize=False) to reduce distributional asymmetry without truncating observed values.

The transformation is defined as:


$$y_{i}^{\mathrm{(\lambda )}}=\{\begin{array}{cc} \frac{{\left(y_{i}+1\right)}^{\lambda }-1)}{\lambda } & \text{if }\lambda \neq 0,y_{i}\geq 0\\ \log\left(y_{i}+1\right) & \text{if }\lambda =0,y_{i}\geq 0\\ -\frac{{\left(-y_{i}+1\right)}^{2-\lambda }-1)}{2-\lambda } & \text{if }\lambda \neq 2,y_{i}<0\\ -\log\left(-y_{i}+1\right) & \text{if }\lambda =2,y_{i}<0 \end{array}$$

where the optimal parameter λ is estimated via maximum likelihood for each variable independently​. Post-transformation verification via z-scores confirmed that no athlete (0 of 21, 0%) exhibited any variable with |z|>3, indicating substantial reduction in outliers without data loss and confirming that the winsorization and power-transformation steps effectively normalized the distribution without residual extreme values requiring further review.

Fifth, robust scaling was applied using scikit-learn’s RobustScaler (Huber, 1964), which standardizes features based on the median and interquartile range rather than mean and standard deviation:


$$z_{ij}=\frac{x_{ij}-\text{median}(x_{j})}{\text{IQR}(x_{j})}$$

where IQR represents Q_3_−Q_1_. RobustScaler was selected over StandardScaler due to its superior performance on transformed data exhibiting residual non-normality, ensuring that all variables contribute equitably to distance-based algorithms (K-Means, Ward linkage) and Principal Component Analysis, independent of their original measurement scales.

### 2.4 Dimensionality reduction

Two complementary dimensionality reduction techniques were employed for exploratory structure visualization. Principal Component Analysis (PCA) (Jollife and Cadima, 2016) was fitted to the scaled matrix with n_components=2, capturing the primary orthogonal directions of variance in the high-dimensional phenotypic space. Component loadings and explained variance ratios were recorded for interpretation. t-distributed Stochastic Neighbour Embedding (t-SNE) (Van Der Maaten and Hinton, 2008) was implemented with a dynamically adjusted perplexity parameter to accommodate small sample sizes. The perplexity value was computed according to:


$$\text{perplexity}=\{\begin{array}{cc} 5 & \text{if }n<15\\ 10 & i\text{f }15\leq n<30\\ \min\left(30,n-1\right) & \text{if }n\geq 30 \end{array}$$

For the present sample (n=21), perplexity was set to 10. Additional configuration parameters included init=‘pca’ for PCA-based initialization to enhance convergence stability, learning_rate=‘auto’ for adaptive modern adjustment, and random_state=42 for computational reproducibility. Importantly, PCA and t-SNE projections were employed exclusively for exploratory visualization of cluster structure in reduced-dimensional space and were not incorporated as predictor variables in subsequent analytical stages. K-Means and hierarchical clustering were performed on the full scaled feature matrix to preserve complete phenotypic information, consistent with best practices when retained PCA variance in two components is insufficient (<20%) to represent the original variable space (Ding and He, 2004).

### 2.5 Clustering algorithms and internal validation

K-Means clustering (MacQueen, 1967) was applied to the scaled feature matrix, with the optimal number of clusters (*k*) determined through convergence analysis of three complementary internal validation metrics. The elbow method evaluated within-cluster sum of squares (inertia) across *k=1-9*, identifying the inflection point where marginal variance reduction diminishes. Silhouette (Rousseeuw, 1987) coefficient analysis (*k=2-9*) quantified cluster cohesion and separation, with higher values indicating superior clustering quality. The Davies-Bouldin index (Davies and Bouldin, 1979) (*k=2-9*) assessed the ratio of within-cluster to between-cluster distances, with lower values reflecting more compact and well-separated clusters. Final *k* was selected as the value maximizing the silhouette coefficient, subject to override on convergent biological-interpretability grounds when justified. All K-Means implementations employed *random_state=42* to ensure reproducibility. Hierarchical clustering was performed using agglomerative clustering with Ward linkage (J. H. Ward, 1963), which minimizes within-cluster variance at each merge step. The resulting dendrogram visualized Euclidean distances between athletes as a function of hierarchical structure. To enable direct algorithmic comparison, the number of clusters extracted from the hierarchical solution was matched to the optimal *k* identified via K-Means. Cluster concordance between methods was quantified using the Adjusted Rand Index (*ARI*) (Hubert and Arabie, 1985), where *ARI=1* indicates perfect agreement and *ARI=0* reflects chance-level similarity.

### 2.6 Statistical characterization of clusters

For each original numeric variable, explicitly excluding synthetic features (PCA/t-SNE components, cluster labels) and performance variables, formal inferential testing was conducted to assess inter-cluster differences. Parametric assumptions were verified through Shapiro-Wilk normality tests (Shapiro and Wilk, 1965) applied to each cluster independently (H_0_: normal distribution; rejection threshold *p<0.05*) and Levene’s test (Levene, 1960) for homogeneity of variance across clusters (H_0_: equal variances; rejection threshold *p<0.05*). When both assumptions were satisfied, one-way ANOVA (Fisher, 1992) was employed to test the null hypothesis of equal means, yielding *F-statistics* and associated *p-values*. For variables violating normality or homoscedasticity assumptions, the non-parametric Kruskal-Wallis H-test (Kruskal and Wallis, 1952) was substituted. Given the small sample size typical of elite athlete cohorts (*n=21*), violations of parametric assumptions were frequently observed, necessitating widespread application of non-parametric alternatives to ensure statistical validity. Statistically significant ANOVA and Kruskal-Wallis results (p < 0.05) were followed by Tukey’s HSD (Tukey, 1949) and Dunn’s (Dunn, 1964) *post-hoc* pairwise comparisons, respectively, to identify specific inter-cluster differences while controlling the family-wise error rate.

Descriptive statistics (mean, standard deviation, minimum, maximum) were computed per cluster for all original variables, with summary tables formatted in “*Mean ± SD*” notation accompanied by *p-values* and statistical test identification to conform with publication standards. Visualization strategies included granular violin plots with embedded quartile lines and superimposed swarmplots to simultaneously convey distributional shape, central tendency, and individual data point positioning. Additionally, a 2×3 panel of box-and-whisker plots was generated for the six variables exhibiting the lowest *p-values*, facilitating comparative assessment of effect sizes and outlier patterns. Radar charts were constructed using MinMaxScaler normalization (range [0.1, 0.9]) applied to the top six discriminant variables, integrating athlete rosters and cluster-specific descriptive statistics within each radial panel. To control the family-wise error rate arising from simultaneous testing across the full variable set, Benjamini-Hochberg False Discovery Rate (FDR) correction was applied to all raw p-values obtained from ANOVA and Kruskal-Wallis tests (Benjamini and Hochberg, 1995). Adjusted p-values ( $p_{\text{adj}}$) were computed using the *multipletests* function from the *statsmodels* library.

### 2.7 Discriminant variable importance and classification rule extraction

To identify variables most influential in driving cluster separation, a Random Forest Classifier (Breiman, 2001) (*n_estimators=100*, *random_state=42*) was trained with K-Means assignments as the target variable and all original numeric variables, excluding synthetic features and performance variables, as predictors. Variable importance scores were calculated as mean Gini impurity reduction across all decision trees, with the top 15 variables ranked and exported for subsequent analysis. To assess the stability of this ranking under the present high-dimensional, small-sample conditions, a bootstrap resampling procedure was additionally conducted (B = 1000 resamples of the n=21 sample, with the Random Forest re-fitted and feature importance recomputed at each iteration), quantifying the proportion of the original top-15 variables retained in the top-15 ranking across resamples. To generate human-interpretable classification rules, a shallow Decision Tree Classifier (Breiman et al., 2017) (*max_depth=3*) was trained exclusively on the top 15 discriminant variables. The dataset was partitioned via stratified random sampling into training (66.7%, n≈14) and independent test (33.3%, n≈7) subsets, with stratification ensuring proportional cluster representation in both partitions (*random_state=42*). The shallow depth constraint was intentionally imposed to prevent overfitting and enhance clinical interpretability by limiting rule complexity. Model performance was evaluated through training accuracy, test accuracy, and a confusion matrix computed on the held-out test set.

An individual athlete verification table was constructed containing athlete identifiers, true cluster assignments, predicted cluster assignments, classification accuracy (Boolean), and dataset partition labels (Train/Test). This table was sorted by accuracy status with misclassifications prioritized at the top to facilitate rapid error pattern identification. Tree visualization parameters were configured to enhance readability. Given the small test partition (*n=7*), Clopper-Pearson exact binomial 95% confidence intervals were computed for both training and test accuracy estimates to quantify uncertainty inherent to limited-sample validation (Clopper and Pearson, 1934). A one-tailed binomial test against chance-level performance (p_0_=1/3, corresponding to random classification across three clusters) was additionally computed to assess whether classification accuracy exceeded expectation under a null model of random assignment.

### 2.8 Software implementation and computational reproducibility

All analyses were implemented in Python 3.12.3 utilizing the following libraries: pandas (data manipulation), numpy (numerical operations), matplotlib and seaborn (visualization), scikit-learn (preprocessing, clustering, classification, model evaluation metrics), scipy (statistical hypothesis testing, hierarchical clustering), statsmodels (Tukey HSD *post-hoc* comparisons), and openpyxl (Excel^®^ input/output operations). To ensure computational reproducibility, the random seed was fixed globally at 42 (*random.seed(42)*, *np.random.seed(42)*) for all stochastic procedures. Analysis outputs were systematically exported to Excel^®^ file across dedicated worksheets. Complete code and de-identified data are available from the corresponding author upon reasonable request.

## 3 Results

### 3.1 Sample characteristics

The final analytical sample comprised 21 elite flatwater canoeists (12 males, 9 females) with mean age 20 ± 4.4 years (range: 15 – 36), height 170.8 ± 10.5 cm (range: 153 – 190), and body weight 70.6 ± 10.5 kg (range: 55.1 – 87.3). Body composition analysis revealed mean body fat percentage of 15.7 ± 4.8%, with male athletes exhibiting significantly lower adiposity (12.1 ± 2.3%) compared to female athletes (20.5 ± 2.4%; p < 0.001, Mann-Whitney U). Statistically significant sex differences were also observed in age, height, and body weight (all p < 0.05; **Table 1**), confirming the mixed-sex phenotypic composition of the cohort and justifying the *post-hoc* interpretive treatment of sex in subsequent cluster characterization (Section 3.2.3). All participants demonstrated competition experience at national or international level, with laboratory assessments capturing performance during peak training phases of the competitive season. Descriptive statistics in **Table 1** reflect raw, unprocessed values prior to the preprocessing pipeline described in Section 2.3; minor discrepancies with cluster-level means are attributable to Winsorization of one age outlier (Section 2.3).

**Table 1:** Baseline anthropometric and body composition characteristics of the total sample and by sex.

Variable	Mean ± SD	Male (n=12)	Female (n=9)	p-value
Age (years)	20.0 ± 4.4	21.67 ± 5.09	17.78 ± 1.99	0.018 *
Height (cm)	170.8 ± 10.5	177.56 ± 6.62	161.83 ± 7.42	0.001 *
Weight (kg)	70.6 ± 10.5	76.96 ± 7.81	62.06 ± 7.08	0.002 *
Body fat (%)	15.7 ± 4.8	12.10 ± 2.33	20.49 ± 2.35	<0.001 *

*p < 0.05 (two-tailed). Statistically significant sex differences were observed across all four variables. Values expressed as Mean ± SD. Between-sex comparisons conducted via Mann-Whitney U test.

### 3.2 Cluster structure and internal validation

#### 3.2.1 Optimal cluster determination

Convergent analysis of three internal validation metrics identified three clusters as the optimal phenotypic segmentation solution (**Figure 2**). The elbow method revealed diminishing marginal returns in within-cluster variance reduction beyond k=3, with inertia stabilizing from 2359 (k=2) to 2184.7 (k=3) to 2029.6 (k=4). Silhouette coefficient analysis identified k=2 as the value with highest cohesion-separation balance (0.110); k=3 exhibited a score of 0.056, declining to 0.039 at k=4. Although k=2 maximizes the silhouette metric in isolation, k=3 was selected based on convergent evidence with the elbow inflection point and greater biological interpretability, as a two-cluster solution collapses intra-sex neuromuscular variation into a single male group. The modest Silhouette coefficient (0.056) reflects the inherent homogeneity of an elite cohort, where phenotypic gradations are subtle rather than categorical. Analogously low silhouette values (<0.50) have been reported in prior clustering studies of sport-specific movement patterns, where biological signal is distributed across high-dimensional continuous gradients rather than discrete categories (Mausehund et al., 2025). The Davies-Bouldin index exhibited a monotonically decreasing trajectory from k=3 (DB = 2.265) through k=9 (DB = 1.401), a pattern consistent with the known behavior of centroid-based validity indices under high-dimensional, small-sample conditions (n=21, p=281), where increasing k mechanistically reduces within-cluster scatter by construction without reflecting genuine cluster structure improvement. Consequently, the DB index was retained as a descriptive validity measure but did not contribute independent discriminative evidence for k selection. The optimal k=3 solution was determined by convergent evidence from the elbow inflection point and the silhouette coefficient, which respectively capture global inertia reduction and local cohesion-separation balance under the present analytical constraints. It should be acknowledged that the choice of k=3 over the silhouette-maximizing k=2 rests on a pragmatic tie-break between statistical and biological criteria under conditions where no internal validation index provides unambiguous guidance; this decision remains provisional pending replication.

**Figure 2:**
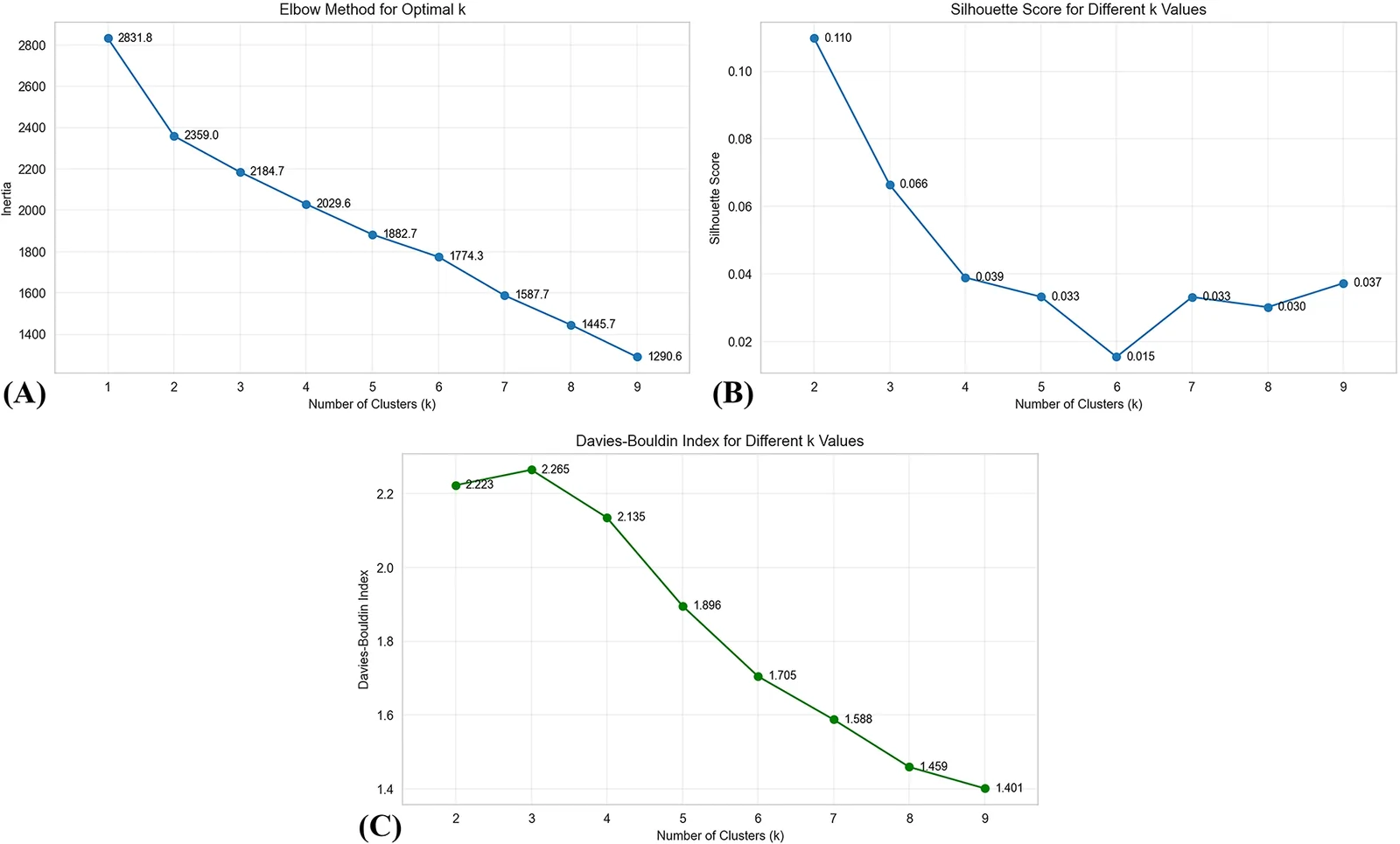
Three-panel figure showing **(A)** Elbow plot (inertia vs. k), **(B)** Silhouette scores vs. k, **(C)** Davies-Bouldin index vs. k.

#### 3.2.2 Cluster composition and algorithmic concordance

K-Means clustering partitioned the sample into three phenotypic groups: Cluster 0 (Moderate-Neuromuscular Male Phenotype, n=4, 19.0%), Cluster 1 (Female-Dominant Phenotype, n=10, 47.6%), and Cluster 2 (High-Neuromuscular Male Phenotype, n=7, 33.3%). These designations are derived from the *post-hoc* sex distribution analysis (Section 3.2.3) and the neuromuscular stratification pattern documented in Sections 3.2.4-3.2.8; they replace performance-based labels to avoid conflation of sexual dimorphism with competitive level. Hierarchical clustering using Ward linkage yielded analogous group structures, with the dendrogram revealing three major branches corresponding to distinct Euclidean distance thresholds in the scaled feature space (**Figure 3a**). The dendrogram exhibits clear hierarchical organization with fusion heights separating at approximately 18–20 Euclidean distance units for the three-cluster solution, indicating well-defined phenotypic boundaries.

**Figure 3:**
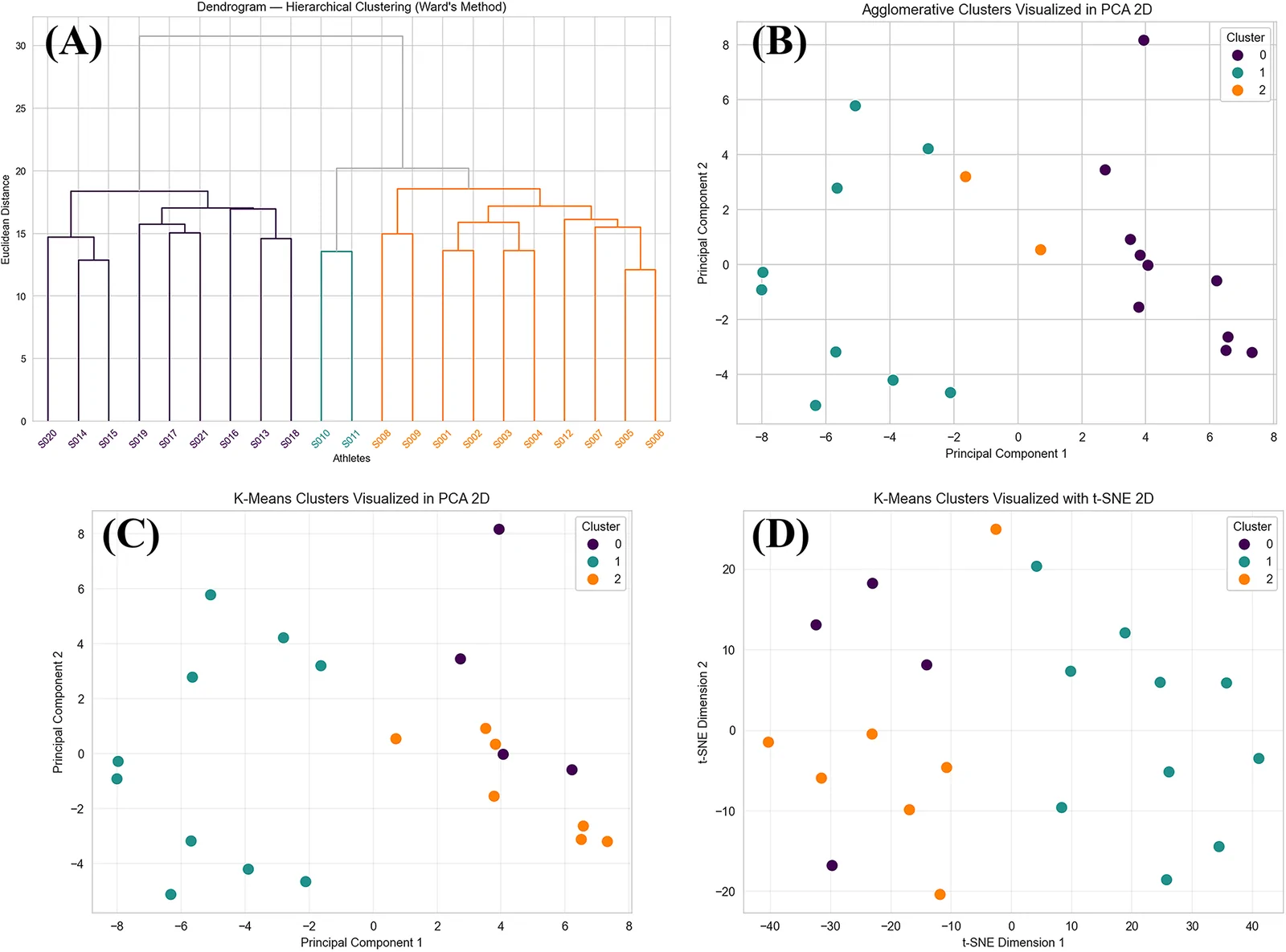
Cluster validation and dimensionality reduction visualization. **(A)** Dendrogram from Ward hierarchical clustering showing three branches at ≈18–20 Euclidean distance units (Clusters 0=purple, 1=cyan, 2=orange). **(B)** Agglomerative cluster assignments in PCA space. **(C)** K-Means clusters projected onto PC1-PC2: Cluster 0 (purple, n=4), Cluster 1 (cyan, n=10), Cluster 2 (orange, n=7). **(D)** t-SNE projection (perplexity=10) revealing enhanced non-linear separation across clusters.

Algorithmic concordance between K-Means and hierarchical methods was quantified via Adjusted Rand Index (ARI = 0.591), indicating moderate agreement beyond chance expectation and confirming reasonable consistency of the three-cluster structure across different optimization criteria. Visual inspection of cluster distributions in reduced-dimension spaces revealed phenotypic separation patterns (**Figures 3b-d**). Principal Component Analysis captured 28.36% of total variance in the first two components (PC1: 19.36%, PC2: 9.00%), with limited variance explained reflecting the high-dimensional complexity of the 281-variable phenotypic space. PC2 contributes a modest additional 9.00% of variance beyond PC1, together explaining less than a third of total phenotypic variability, a pattern consistent with the high-dimensional, small-sample setting (n=21, p=281) in which no small subset of linear components can be expected to capture the majority of variance. In the PCA projection (**Figure 3c**), Cluster 1 (Female-Dominant Phenotype, n=10) exhibited the broadest spatial distribution across both PC1 and PC2, spanning from negative to positive PC1 values, suggesting greater phenotypic heterogeneity within this numerically dominant group. Cluster 0 (n=4) and Cluster 2 (n=7) demonstrated more compact positioning, with Cluster 0 occupying the upper-right quadrant (positive PC1, positive PC2) and Cluster 2 concentrated in the lower-right region (positive PC1, negative PC2), indicating distinct phenotypic profiles despite partial overlap along PC1. t-SNE projections (perplexity=10) revealed enhanced cluster separation in the non-linear embedding space (**Figure 3d**), with Cluster 0 forming a compact group in the left-centre region, Cluster 1 displaying wider dispersion across the right and lower quadrants, and Cluster 2 positioned primarily in the lower-centre area. The improved visual separation in t-SNE compared to PCA suggests that phenotypic differences involve non-linear relationships not fully captured by linear dimensionality reduction, supporting the complementary use of both techniques for exploratory cluster visualization.

#### 3.2.3 Sex distribution across clusters

Sex distribution exhibited marked stratification across the three phenotypic clusters (χ²=18.90, p<0.001; **Table 2**). Cluster 1 demonstrated disproportionate female representation (n=9, 90.0%), with only one male athlete (n=1, 10.0%). In contrast, Clusters 0 and 2 were exclusively male (n=4, 100%; n=7, 100%, respectively). This pronounced sex-cluster association indicates that the tripartite phenotypic architecture reflects, to a substantial degree, sexual dimorphism in neuromuscular capacity, body composition, and anthropometric characteristics. Accordingly, Cluster 1 is designated as the Female-Dominant Phenotype, a label that describes its demographic composition without implying inferiority of competitive level relative to the male-dominant clusters. The phenotypic differences observed between Cluster 1 and Clusters 0/2 should be interpreted as normative biological variation between sexes rather than as a performance gradient within a homogeneous population. Specifically, the elevated body fat percentage (19.5 ± 3.6% vs. 12.2% ± 2.3% in Cluster 0 and 12.6% ± 1.9% in Cluster 2), reduced muscle mass (47.8 ± 5.4% vs. 66.2% ± 5.8% and 64.5% ± 5.8%), and attenuated neuromuscular capacity observed in Cluster 1 may reflect normative sex-specific physiological differences rather than training deficits or developmental limitations. Relative maximal strength (*1RM/kg* bench press) was correspondingly lower in Cluster 1 (1.2 ± 0.1 vs. 1.6 ± 0.2 and 1.5 ± 0.2), consistent with normative sex-specific differences in absolute force production capacity (Hunter, 2014; Staron et al., 2000).

**Table 2:** Sex distribution across phenotypic clusters.

Cluster	N	Male	Female	% Female
0	4	4 (100%)	0 (0%)	0.0%
1	10	1 (10%)	9 (90%)	90.0%
2	7	7 (100%)	0 (0%)	0.0%
Total	21	12 (57%)	9 (43%)	42.9%

Cluster 1 exhibits disproportionate female representation relative to sample composition (90% vs. 43% overall, Fisher’s exact test p<0.001). Clusters 0 and 2 are exclusively male Chi-square test of independence: χ²=18.90, p<0.001, indicating statistically significant association between sex and cluster assignment.

Female athletes typically exhibit higher essential fat stores (10-13% vs. 2-5% in males) and lower lean mass proportions due to hormonal and constitutional factors, patterns consistent with the Cluster 1 profile. The complete absence of female athletes in Clusters 0 and 2 precludes definitive separation of sex effects from performance-related phenotypic variation within the current analytical framework. Although body-mass normalization was systematically applied to neuromuscular variables, this approach corrects only for absolute force scaling and does not account for sex-dependent differences in muscle fiber composition, hormonal milieu, or biomechanical lever systems that influence relative force production capacity. Future research with larger sex-stratified cohorts is required to determine whether distinct performance phenotypes exist within each sex, or whether the observed tripartite structure primarily reflects sexual dimorphism superimposed on a performance continuum.

#### 3.2.4 Discriminant variable importance

Random Forest Classifier analysis quantified discriminant power across the 281-variable phenotypic space via mean Gini impurity reduction over 100 decision trees. The top 15 features (**Figure 4**) accounted for 39.4% of cumulative importance, with neuromuscular variables from the canoe-specific FEMD incremental test dominating the hierarchy (75.9% of top-15 cumulative importance). *Fpeak FEMD_INC-G_* exhibited the highest discriminant capacity (importance=0.061), followed by *n°reps FEMD_INC_* (repetitions to failure, 0.033) and *work FEMD_INC-G_* (total mechanical work, 0.032) collectively accounting for 12.6% of total importance; LD muscle contractile properties (*Ts non-paddle*, 0.019) contributed an additional 1.9%. Beyond the top three, non-neuromuscular variables also contributed meaningfully: distance from C7 spinous to fingertip (importance=0.028), basal metabolic expenditure (0.025) and bone mass (0.023), indicating a multi-domain phenotypic architecture underlying cluster discrimination.

**Figure 4:**
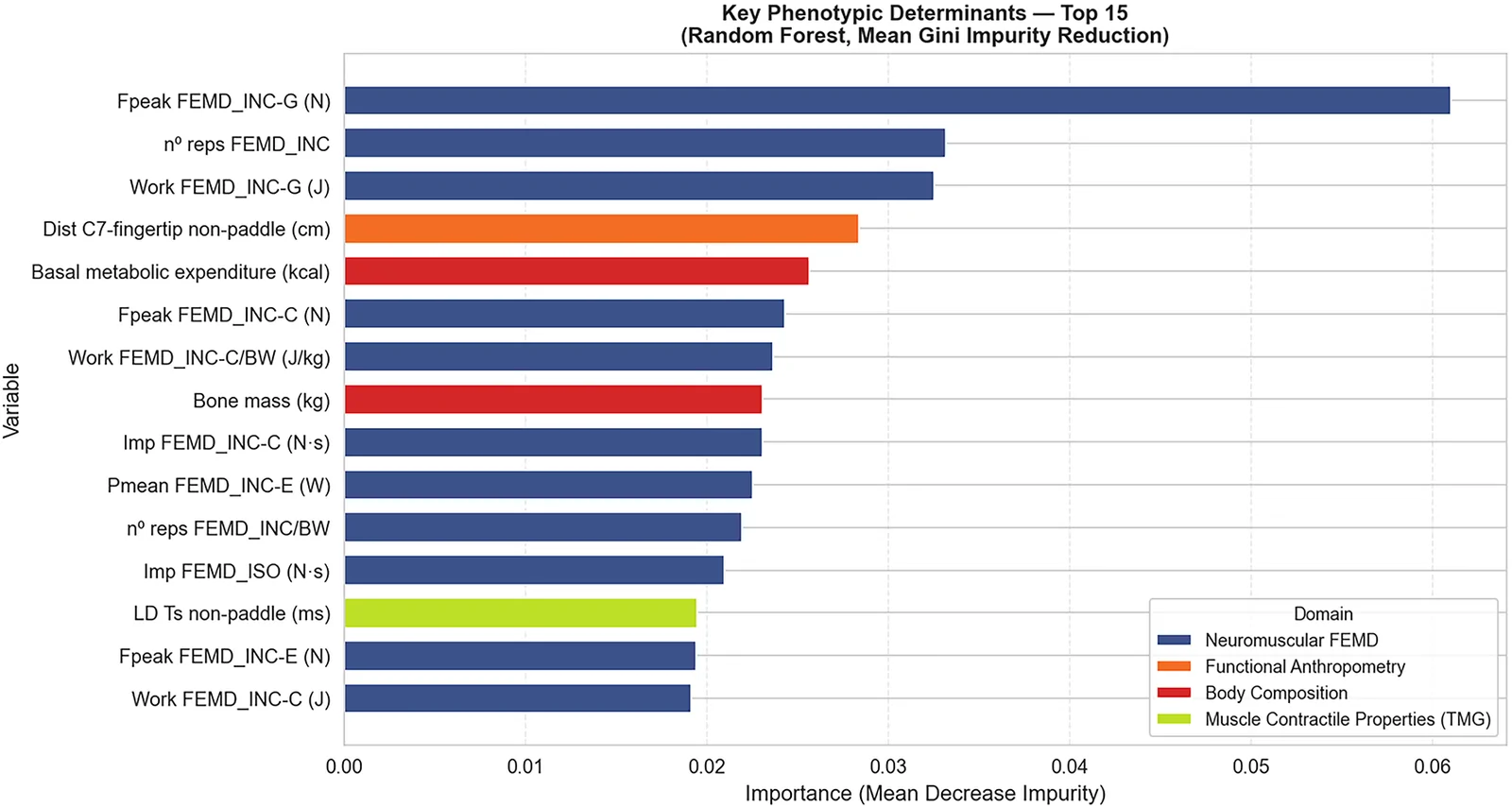
Discriminant variable importance hierarchy for phenotypic cluster separation. Horizontal bar chart depicting top 15 features ranked by Random Forest mean Gini impurity reduction (100 trees, bootstrap aggregation). Fpeak FEMD_INC-G_ demonstrates dominant discriminant capacity (0.061), with neuromuscular variables comprising 75.9% of cumulative importance. Color-coded by domain: neuromuscular (blue), anthropometric-metabolic (orange), body composition (red), functional anthropometry + muscle contractile properties (green).

Beyond these cardinal neuromuscular determinants, the feature importance profile revealed multidimensional stratification mechanisms. Arm length contributed through distance from C7 to fingertip from non-paddle side (0.028, rank 4), basal metabolic expenditure via Kcal (0.025, rank 5), and skeletal structure through bone mass (0.023, rank 8). Additional neuromuscular metrics included Fpeak FEMD_INC-C_ (0.024), Work FEMD_INC-C/kg_ (0.023), Imp FEMD_INC-C_ (0.023), and Pm FEMD_INC-E_ (0.022). Muscle contractile properties manifestation emerged via LD Ts from non-paddle side (0.019, rank 13). Domain-level decomposition of top-15 importance yielded: neuromuscular 75.9%, trunk flexibility 7.1%, metabolic-anthropometric 6.3%, body composition 5.8%, and muscle contractile properties 4.8%, supporting the primacy of sport-specific force production while indicating that elite phenotypic architecture emerges from integrated multisystem determinants rather than isolated physical capacities. Bootstrap resampling (B = 1000) indicated that, on average, only 36.9% (SD = 12.7%; median=40.0%; 95% CI: 13.3%–60.0%) of these top-15 variables were retained in the top-15 ranking across resamples, indicating substantial ranking instability consistent with the high-dimensional, small-sample setting (n=21, p=281).

#### 3.2.5 Statistical characterization of phenotypic clusters

Parametric assumptions were verified through Shapiro-Wilk normality tests and Levene’s homoscedasticity assessment, enabling one-way ANOVA or Kruskal-Wallis application throughout (**Table 3**). Across the full 281-variable battery, inferential testing identified 88 variables with raw p < 0.05; following Benjamini–Hochberg FDR correction, 51 variables retained statistical significance ( $p_{\text{adj}}$ < 0.05). The 15 variables reported in **Tables 3**, **4** were selected on the basis of discriminant importance derived from Random Forest Classifier analysis; 14 of these 15 retained FDR-corrected significance ( $p_{\text{adj}}$ range: 9.49×10^-6^ to 8.00×10^-^³), with *LD Ts from non-Paddle* as the sole exception ( $p_{\text{adj}}$ = 0.105), indicating convergent validity between inferential significance and machine-learning-derived discriminance across neuromuscular and anthropometric domains.

**Table 3:** Statistical characterization of phenotypic clusters across discriminant variables.

Variable	Cluster 0 (n=4)	Cluster 1 (n=10)	Cluster 2 (n=7)	p-value	$p_{\text{adj}}$
Neuromuscular FEMD (Isometric)					
*Imp FEMD_ISO_ (N s)*	2664.6 ± 216.2	1932.7 ± 321.5	3023.0 ± 438.8	< 0.001	0.008
Neuromuscular FEMD (Incremental Resistance)					
*n° reps FEMD_INC_*	13.8 ± 1.5	8.2 ± 1.0	13.7 ± 1.4	< 0.001	0.00001
*n°reps FEMD_INC/Kg_*	0.182 ± 0.03	0.134 ± 0.01	0.177 ± 0.01	< 0.001	0.008
Neuromuscular FEMD — Incremental: Concentric (C)					
*Fpeak FEMD_INC-C_ (N)*	55.9 ± 11.5	34.2 ± 3.2	58.2 ± 8.9	< 0.001	0.00598
*Work FEMD_INC-C_ (J)*	5182.7 ± 1748.3	1900.6 ± 541.4	5333.1 ± 1823.6	< 0.001	0.00203
*Work FEMD_INC-C/Kg_ (J/kg)*	65.6 ± 20.8	30.3 ± 7.1	67.8 ± 18.8	< 0.001	0.00598
*Imp FEMD_INC-C_ (N s)*	5006.9 ± 1909.0	2086.2 ± 710.9	4875.7 ± 1531.8	< 0.001	0.00531
Neuromuscular FEMD — Incremental: Eccentric (E)					
*Fpeak FEMD_INC-E_ (N)*	50.2 ± 10.5	32.2 ± 3.1	54.5 ± 6.4	< 0.001	0.00013
*Pm FEMD_INC-E_ (W)*	268.2 ± 55.3	159.1 ± 29.5	264.5 ± 44.0	< 0.001	0.00138
Neuromuscular FEMD — Incremental: Global (G)					
*Fpeak FEMD_INC-G_ (N)*	56.7 ± 10.6	34.6 ± 3.2	59.2 ± 8.5	< 0.001	0.00598
*Work FEMD_INC-G_ (J)*	8510.3 ± 3433.7	3062.3 ± 837.0	8358.2 ± 2948.9	< 0.001	0.00349
Body Composition					
*Bone mass (kg)*	3.4 ± 0.3	2.6 ± 0.2	3.4 ± 0.3	< 0.001	0.008
*BMR (Kcal)*	4307.2 ± 437.8	2832.6 ± 384.7	4219.4 ± 376.0	< 0.001	0.00598
Functional Anthropometry					
*Dis C7 non-paddle (cm)*	93.8 ± 2.8	83.0 ± 3.8	95.4 ± 1.9	< 0.001	0.00598
Muscle Contractile Properties (TMG)					
*LD Ts non-paddle*	162.6 ± 5.5	196.1 ± 81.2	118.2 ± 27.3	0.025	0.10466

Parametric assumptions verified via Shapiro-Wilk and Levene’s tests; ANOVA applied where assumptions were met, Kruskal-Wallis otherwise (see **Table 4**). Data presented as Mean ± SD. Variables organized by functional domain. $p_{\text{adj}}$ Benjamini-Hochberg FDR-corrected p-value.

**Table 4:** *Post-hoc* pairwise comparisons of neuromuscular and anthropometric characteristics across the identified phenotypic clusters.

Variable	Model	Test	C0-C1	C0-C2	C1-C2
Neuromuscular FEMD (Isometric)					
*Imp FEMD_ISO_*	Kruskal-Wallis	Dunn	0.0674	0.3341 ns	0.0008**
Neuromuscular FEMD (Incremental Resistance)					
*n° reps FEMD_INC_*	ANOVA	Tukey	<0.001***	0.9988 ns	<0.001***
*n°reps FEMD_INC/Kg_*	Kruskal-Wallis	Dunn	0.0099**	0.9520 ns	0.0026**
Neuromuscular FEMD — Incremental: Concentric (C)					
*Fpeak FEMD_INC-C_*	Kruskal-Wallis	Dunn	0.0086**	0.8794 ns	0.0013**
*Work FEMD_INC-C_*	ANOVA	Tukey	0.0016**	0.9822 ns	0.0002***
*Work FEMD_INC-C/Kg_ (J/kg)*	Kruskal-Wallis	Dunn	0.0086**	0.8794 ns	0.0013**
*Imp FEMD_INC-C_ (N s)*	ANOVA	Tukey	0.0032**	0.9854 ns	0.0009**
Neuromuscular FEMD — Incremental: Eccentric (E)					
*Fpeak FEMD_INC-E_ (N)*	ANOVA	Tukey	0.0002***	0.5093 ns	<0.001***
*Pm FEMD_INC-E_ (W)*	ANOVA	Tukey	0.0006**	0.9881 ns	0.0001***
Neuromuscular FEMD — Incremental: Global (G)					
*Fpeak FEMD_INC-G_ (N)*	Kruskal-Wallis	Dunn	0.0086**	0.8794 ns	0.0013**
*Work FEMD_INC-G_ (J)*	ANOVA	Tukey	0.0021**	0.9938 ns	0.0005**
Body Composition					
*Bone mass (kg)*	Kruskal-Wallis	Dunn	0.0081**	0.9667 ns	0.003**
*BMR (Kcal)*	Kruskal-Wallis	Dunn	0.0045**	0.8794 ns	0.0023**
Functional Anthropometry					
*Dis C7 non-paddle (cm)*	Kruskal-Wallis	Dunn	0.0156*	0.6482 ns	0.0008**
Muscle Contractile Properties (TMG)					
*LD Ts non-paddle*	Kruskal-Wallis	Dunn	0.6823	0.1307 ns	0.0229*

Significance: *p<0.05, **p<0.01, ***p<0.001, ns, not significant. Omnibus statistical significance was determined via ANOVA or Kruskal-Wallis tests, depending on data distribution. Specific inter-cluster differences were subsequently evaluated using Tukey’s Honestly Significant Difference (HSD) for parametric data and Dunn’s test for non-parametric data.

The emergent phenotypic architecture revealed a tripartite structure characterised by systematic neuromuscular stratification. *Post-hoc* comparisons (Tukey HSD and Dunn; **Table 4**) demonstrated that Cluster 1 exhibited significantly attenuated neuromuscular capacity relative to both Cluster 0 and Cluster 2 across all incremental FEMD-derived metrics (all pairwise p ≤ 0.010), manifesting as ≈40% reductions in *Fpeak FEMD_INC-G_* (34.6 ± 3.2 N vs. 56.7 ± 10.6 N and 59.2 ± 8.5 N, respectively), ≈63% reductions in *Work FEMD_INC-G_* (3062.3 ± 837.0 J vs. 8510.3 ± 3433.7 J and 8358.2 ± 2948.9 J), and ≈40% reductions in *n° reps FEMD_INC_* (8.2 ± 1.0 vs. 13.8 ± 1.5 and 13.7 ± 1.4). The isometric domain presented a partially divergent pattern: Cluster 2 demonstrated superior *Imp FEMD_ISO_* relative to Cluster 1 (3023.0 ± 438.8 vs. 1932.7 ± 321.5 N·s; p = 0.001), whereas the Cluster 0–Cluster 1 difference did not reach statistical significance (2664.6 ± 216.2 vs. 1932.7 ± 321.5 N·s; p = 0.067). Clusters 0 and 2 demonstrated statistically equivalent profiles across all 15 pairwise comparisons (all p > 0.05; range: 0.131–0.999), indicating complete functional equivalence between these two phenotypes despite their numerical distinction in the clustering algorithm.

Body composition and metabolic variables corroborated the neuromuscular stratification pattern. Cluster 1 exhibited significantly reduced bone mass relative to Clusters 0 and 2 (bone: 2.6 ± 0.2 kg vs. 3.4 ± 0.3 kg and 3.4 ± 0.3 kg; Dunn p = 0.008 and p = 0.003, respectively), consistent with lower skeletal density normatively associated with the female-dominant composition of this cluster. Estimated energy expenditure (Kcal) similarly differentiated Cluster 1 from both male-dominant clusters (2832.6 ± 384.7 vs. 4307.2 ± 437.8 and 4219.4 ± 376.0 kcal; Dunn p = 0.005 and p = 0.002), a pattern that reflects the combined effect of lower muscle mass and body weight characteristic of this phenotype. Both variables demonstrated complete equivalence between Clusters 0 and 2 (Bone: p = 0.967; Kcal: p = 0.879).

Functional anthropometry and muscle contractile properties variables presented a distinct discriminant profile. *Dis C7 non-paddle* confirmed significantly reduced arm length in Cluster 1 relative to Clusters 0 and 2 (83.0 ± 3.8 cm vs. 93.8 ± 2.8 cm and 95.4 ± 1.9 cm; Dunn p = 0.016 and p < 0.001, respectively), while Clusters 0 and 2 remained equivalent (p = 0.648). *LD Ts non-paddle* yielded an inverse discriminant pattern, with Cluster 2 exhibiting the lowest values relative to Cluster 1 (118.2 ± 27.3 vs. 196.1 ± 81.2; Dunn p = 0.023) — the sole pairwise comparison to reach significance, as neither Cluster 0 vs. Cluster 1 nor Cluster 0 vs. Cluster 2 attained p < 0.05 — however, this variable did not survive FDR correction ( $p_{\text{adj}}$ = 0.105), and the high within-group dispersion in Cluster 1 (SD = 81.2) limits its inferential interpretation. Its inclusion is retained on the basis of RF discriminant importance and its role as a secondary classification node in the Decision Tree analysis (Section 3.2.6).

Distributional analysis of repetitions to voluntary failure (**Figure 5**) confirms systematic stratification. Cluster 1 (female-dominant) demonstrated lower absolute *n° reps FEMD_INC_* (median: 8.0 vs. 14.0 reps in Clusters 0 and 2; **Figure 5a**). Body-weight normalization attenuated but did not eliminate this gap, reflecting residual sex-specific differences in muscle fiber composition, hormonal milieu, and biomechanical leverage that body-mass scaling cannot fully correct. This pattern is consistent with established evidence on sexual dimorphism in sport-specific force production rather than exclusively indicative of training deficits. Body-weight normalization (*n° reps FEMD_INC/kg_*: 0.134 reps in Cluster 1, 0.182 vs. 0.177 reps in Clusters 0 and 2; **Figure 5b**) attenuated but did not eliminate this deficit. The persistence of inter-cluster differences post-normalization indicates that performance stratification transcends body mass effects, implicating intrinsic neuromuscular efficiency or training adaptations. Clusters 0 and 2 exhibited overlapping probability density functions, reinforcing their phenotypic equivalence.

**Figure 5:**
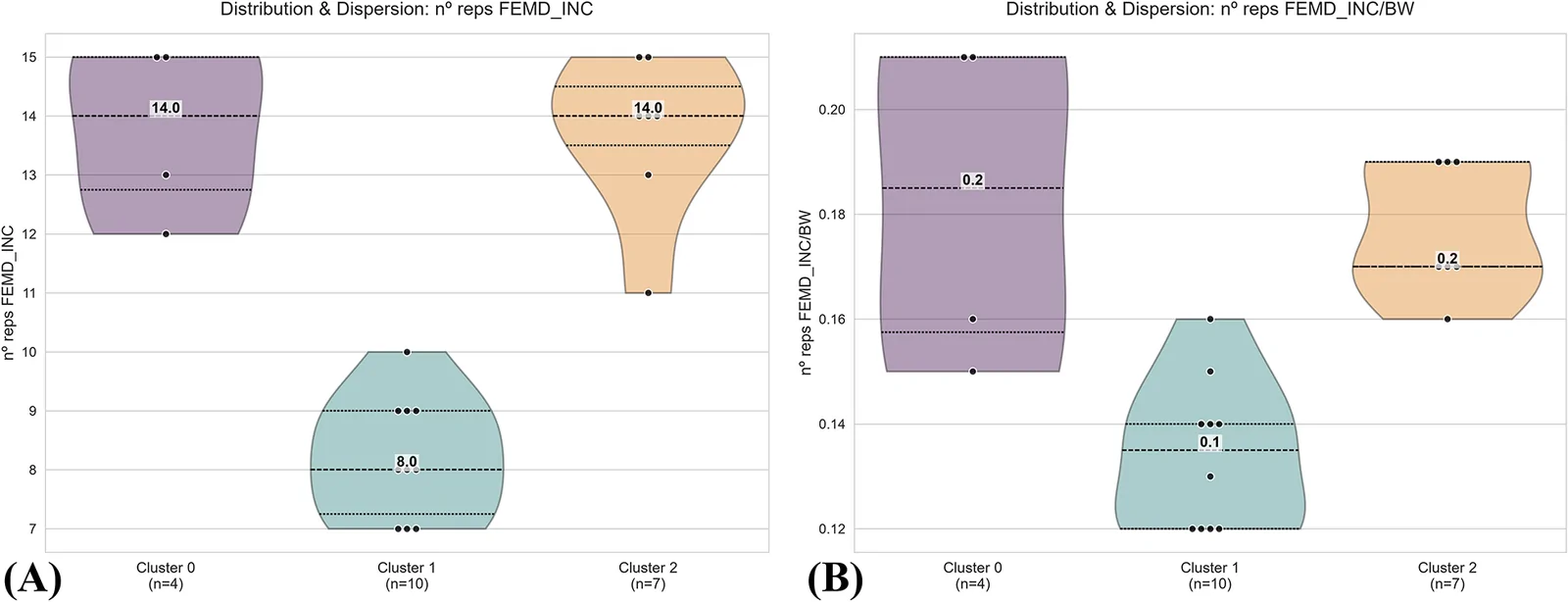
Violin plot analysis of repetitions to voluntary failure. **(A)** Absolute repetitions (*n° reps FEMD_INC_*): Cluster 1 exhibits ~40% reduction (means)/43% reduction (medians) vs. Clusters 0 and 2. **(B)** Body-weight-normalized repetitions (*n° reps FEMD_INC/kg_*): 27% deficit persists post-normalization. Violin width: kernel density; white box: IQR; horizontal line: median; circles: individual data. Cluster 0 (purple, n=4), Cluster 1 (cyan, n=10), Cluster 2 (orange, n=7). ANOVA p<0.001.

Distributional characteristics of the six highest-ranked discriminant variables are visualized in **Figure 6** as a 2×3 panel array of box-and-whisker plots with superimposed individual observations. The six variables displayed are *n° reps FEMD_INC_*, *Fpeak FEMD_INC-E_*, *Pm FEMD_INC-E_*, Work *FEMD_INC-C_*, *Work FEMD_INC-G_*, and *Imp FEMD_INC-C_*, all from the FEMD incremental domain, reflecting their classification as the six lowest raw p-values among the 15 RF-selected variables (all ANOVA; p range: <0.0001 to 0.0003). Visual inspection confirms the systematic positioning of Cluster 1 within the lower quantile ranges across all panels, with minimal —though not entirely absent — distributional overlap relative to Clusters 0 and 2, whose interquartile ranges exhibit substantial convergence across all variables. Among neuromuscular metrics, *n°reps FEMD_INC_* demonstrated the most complete separation, with no overlap between Cluster 1 and the two male phenotypes. The consistent separation pattern across all six incremental FEMD variables, spanning force, power, work, and impulse, reinforces the primacy of sport-specific neuromuscular capacity as the primary axis of phenotypic stratification in this elite cohort.

**Figure 6:**
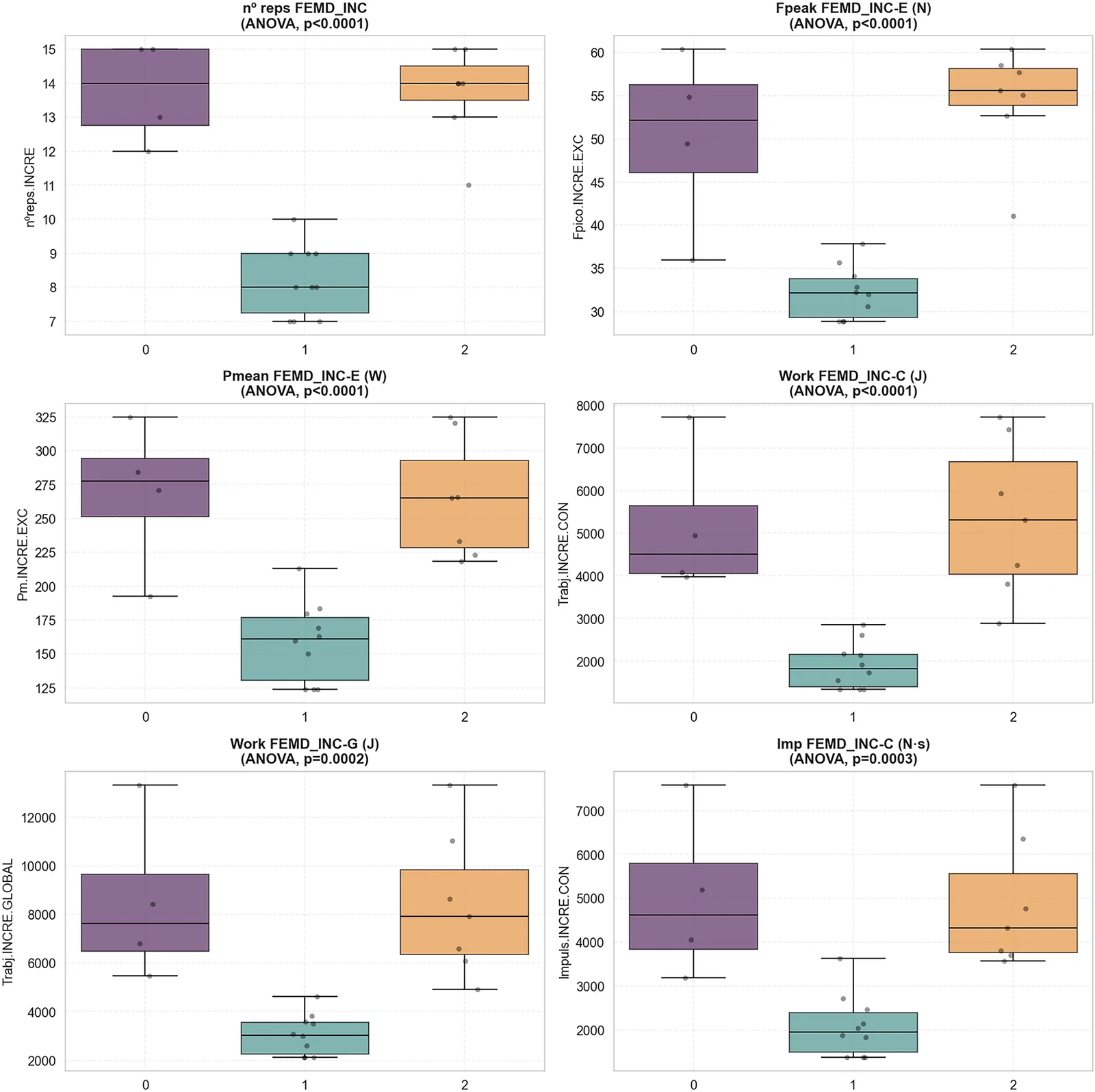
Distributional comparison of top six discriminant variables across phenotypic clusters. Box-and-whisker representations with individual data points (black circles) for Cluster 0 (purple, n=4), Cluster 1 (cyan, n=10), and Cluster 2 (orange, n=7). Horizontal line: median; box boundaries: interquartile range (25th-75th percentiles); whiskers: 1.5×IQR. All variables exhibited p<0.001 via one-way ANOVA with parametric assumption verification.

#### 3.2.6 Cluster stability validation via decision tree classification

To assess cluster robustness and develop a classification framework for prospective athlete phenotyping, a shallow Decision Tree Classifier (*max_depth=3*) was trained exclusively on the top 15 discriminant variables, with stratified partitioning into training (n=14, 66.7%) and independent test (n=7, 33.3%) sets. The model achieved perfect training accuracy (100.0%, 14/14; 95% CI: 76.8%–100.0%) and maintained 71.4% accuracy on held-out data (5/7 correct classifications; 95% CI: 29.0%–96.3%, Clopper-Pearson exact method), with the 28.6-percentage-point generalization gap reflecting expected variance in small-sample external validation rather than systematic overfitting. Although the test accuracy point estimate significantly exceeds chance-level performance (33.3% for three-class random assignment; one-tailed binomial test p=0.045), the wide confidence interval is a direct statistical consequence of the seven-case test partition and precludes strong claims regarding prospective generalization accuracy.

Confusion matrix analysis revealed asymmetric error patterns concentrated at inter-cluster boundaries. Misclassifications involved athlete SP12 (Cluster 0 - 1: Moderate-Neuromuscular Male Phenotype-to-Female Phenotype transition) and athlete SP04 (Cluster 2 - 0: High-Neuromuscular Male Phenotype-to-Moderate-Neuromuscular Male Phenotype), both exhibiting feature values proximal to decision thresholds in high-dimensional space. SP04’s misclassification at the Cluster 2→Cluster 0 boundary is mechanistically consistent with the complete statistical equivalence between these two phenotypes documented across all 15 pairwise comparisons in Section 3.2.5 (all p > 0.05), suggesting that this error reflects genuine phenotypic ambiguity rather than model failure. Critically, all four Cluster 1 athletes (Female-Dominant Phenotype) were correctly classified in the test partition, with no errors at the Cluster 1 - Cluster 2 boundary, consistent with the sex-normative force differences documented in Section 3.2.3. Individual verification (**Table 5**) shows that 90.5% of athletes (19/21) were correctly classified across both partitions, providing preliminary support for cluster stability; model generalizability requires external validation (see Section 4).

**Table 5:** Individual athlete classification verification.

Athlete ID	True cluster	Predicted cluster	Correct	Partition
SP12	0	1	No	Test
SP04	2	0	No	Test
SP18	1	1	Yes	Test
SP19	1	1	Yes	Test
SP07	2	2	Yes	Test
SP16	1	1	Yes	Test
SP13	1	1	Yes	Test
SPXX - Others	[various]	[match]	Yes	Training

Training set (n=14): 100% accuracy. Test set (n=7): 71.4% accuracy. Misclassifications prioritized; training set athletes (n=14) listed collectively.

The interpretable decision tree architecture (**Figure 7**) encodes a parsimonious two-threshold classification algorithm enabling prospective athlete phenotyping without computational infrastructure. The root node partitions on *Fpeak FEMD_INC-E_* ≤ 39.48 N, immediately segregating Cluster 1 (female-dominant phenotype, 42.9% of the training partition) when eccentric force production falls below this threshold, a result attributable to both sex-normative lower absolute force production and any sport-specific training differences within this phenotypic group. This split primarily reflects sex-normative force differences rather than an absolute biomechanical deficiency and should not be interpreted as a universal performance barrier applicable across sexes without recalibration on sex-stratified samples. Athletes exceeding 39.48 N proceed to a secondary bifurcation on *LD Ts* from non-paddle side ≤ 152.05 ms, where values ≤152.05 ms classify to Cluster 2 (High-Neuromuscular Male Phenotype, 35.7% of the training partition) and values >152.05 ms classify to Cluster 0 (Moderate-Neuromuscular Male Phenotype, 21.4% of the training partition). It bears noting that *LD Ts* from non-paddle side was the sole variable among the RF top-15 not surviving Benjamini–Hochberg FDR correction ( $p_{\text{adj}}$ = 0.105; Section 3.2.5); its selection as the secondary DT node reflects RF-derived discriminant importance rather than unconditional inferential significance, and its classification threshold (152.05 ms) should be interpreted accordingly.

**Figure 7:**
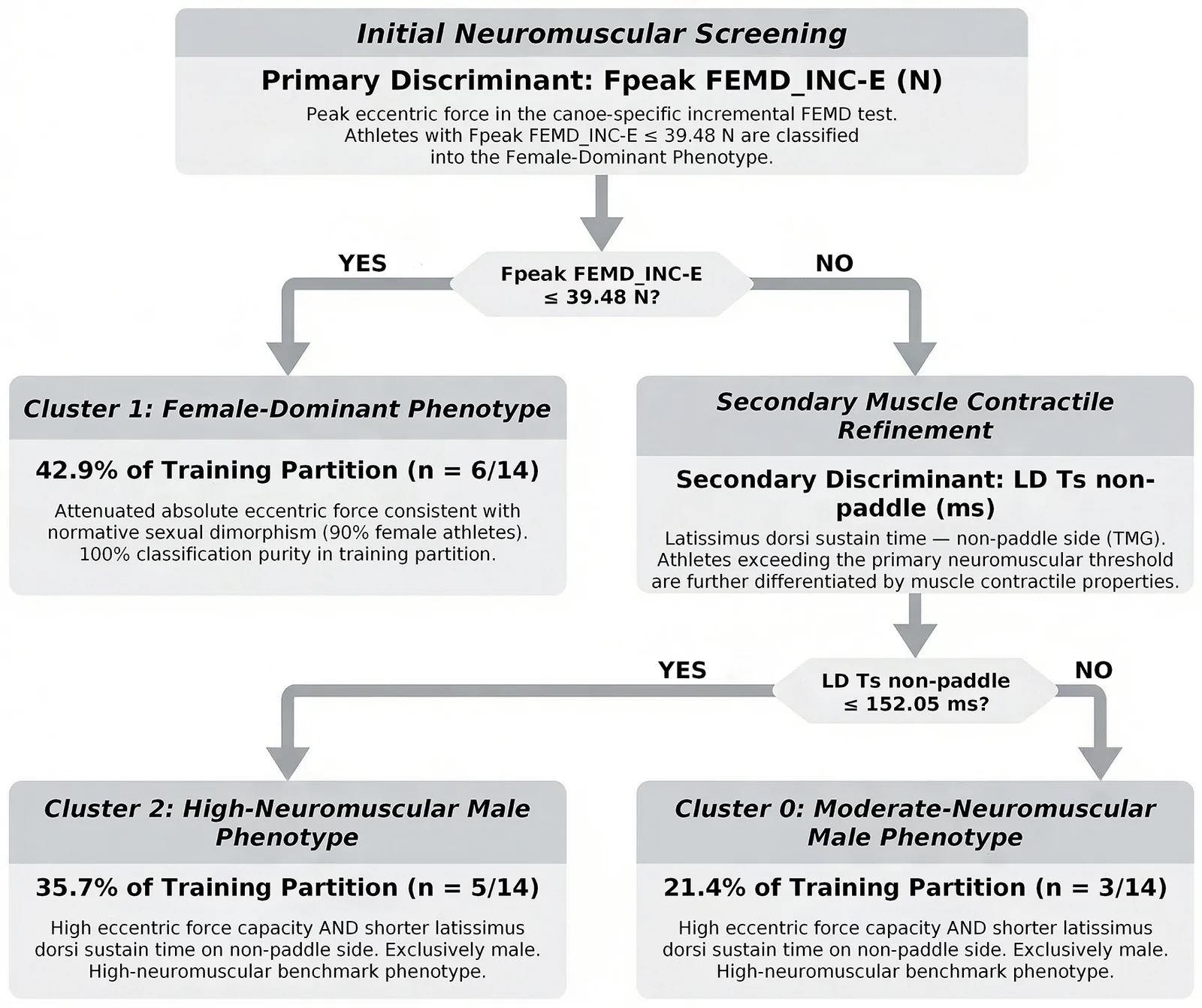
Decision tree explanatory classification structure. The hierarchical architecture reveals a two-stage functional prioritization: (1) Force Production Threshold: The primary split on *Fpeak (*≤ 39.48 N) segregates the Female-Dominant Phenotype (Cluster 1), whose lower absolute force output reflects both normative sexual dimorphism and, secondarily, neuromuscular development stage. This threshold should not be interpreted as a universal performance barrier without sex-stratified recalibration. (2) Muscle contractile properties refinement: Among athletes exceeding the eccentric force threshold (Clusters 0 and 2, exclusively male), the secondary split on LD *Ts* from the non-paddle side differentiates the two male phenotypes based on contractile properties, athletes with shorter sustain time being classified into the High-Neuromuscular Phenotype. This architecture suggests that, within the male phenotypic space, neuromuscular force capacity is a necessary but insufficient condition for elite classification: muscle contractile properties provide the discriminating second criterion.

This hierarchical structure illustrates candidate mechanistic determinants of phenotypic stratification: (1) neuromuscular eccentric capacity serves as the primary discriminant, establishing a foundational force production threshold (39.48 N) that differentiates the Female-Dominant Phenotype from the two male phenotypes—athletes unable to generate eccentric forces exceeding this value exhibit systematically depressed neuromuscular profiles consistent with Cluster 1 characterization; (2) among athletes surpassing this neuromuscular baseline (57.1% of cohort), functional anthropometry becomes the decisive factor, with counter-intuitive directionality wherein lower arm length paradoxically associates with elite status (Cluster 2), potentially reflecting biomechanical efficiency wherein elite athletes achieve superior stroke amplitude for force transmission, maximizing energy transfer along the primary propulsive axis. It should be noted that the eccentric force threshold (39.48 N) is calibrated on a mixed-sex sample and therefore encodes sex-normative differences. Applied prospectively, this threshold should be interpreted in sex-specific contexts: female athletes routinely producing forces near or below this value may nonetheless represent high-performing individuals within their sex-specific performance distribution. A sex-stratified replication is required to establish sex-specific eccentric force thresholds with genuine discriminant validity. The classification framework may inform multiple applied contexts, pending external validation: (1) talent identification, enabling rapid classification of new athletes into established phenotypic profiles via two laboratory metrics (eccentric force + trunk displacement); (2) training individualization, informing cluster-specific programming to address phenotypic deficiencies—Cluster 1 athletes require neuromuscular power enhancement protocols, while Cluster 0 athletes may benefit from trunk stability optimization to reduce excessive lateral displacement; and (3) longitudinal monitoring, detecting phenotypic transitions across training macrocycles via repeated classification. It is noted that the sex composition of the test partition (n=7) was not explicitly reported; given the strong sex-cluster association documented in **Table 2** (Section 3.2.3), interpretation of individual misclassifications (SP12, SP04) should account for the possibility that proximity to decision thresholds reflects sex-related anthropometric characteristics rather than purely performance-based phenotypic ambiguity. The parsimonious two-threshold architecture (effective depth=2) ensures clinical interpretability and deployment feasibility.

#### 3.2.7 Comparative phenotypic profiles via radar chart visualization

Normalized mean values of six discriminant variables were visualized in radar chart format to facilitate intuitive phenotypic comparison across clusters (**Figure 8**). Each radial panel integrated the normalized mean profile (coloured polygon), complete athlete roster, and descriptive statistics summary for key phenotypic determinants.

**Figure 8:**
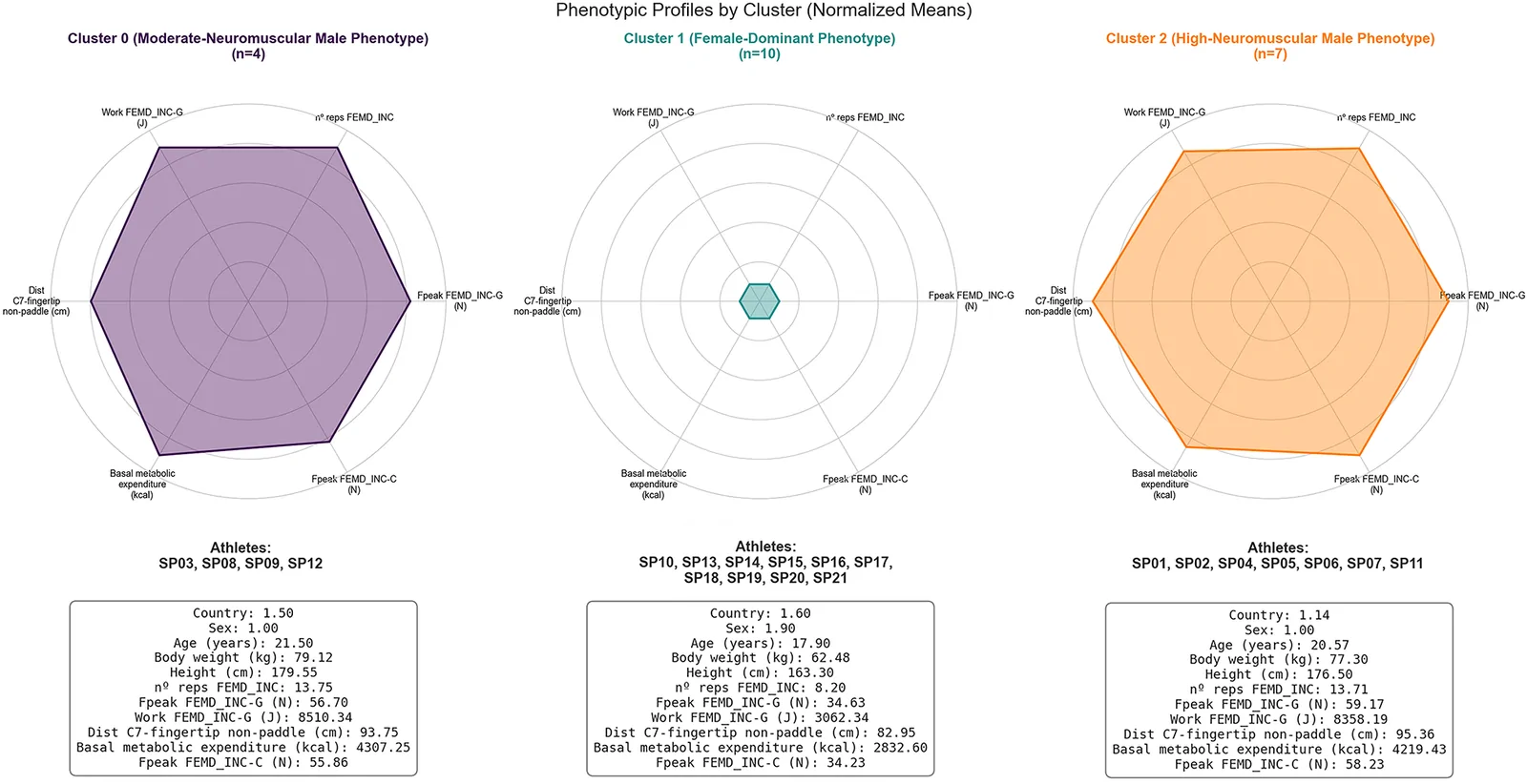
Comparative phenotypic profiles via normalized radar charts. Three-panel visualization depicting normalized mean values (MinMaxScaler range [0.1, 0.9]) for six discriminant variables across phenotypic clusters. Left panel: Cluster 0 (Moderate-Neuromuscular Male Phenotype, n=4, purple polygon)—athletes SP03, SP08, SP09, SP12. Center panel: Cluster 1 (Female-Dominant Phenotype, n=10, cyan polygon)—athletes SP10, SP13, SP14, SP15, SP16, SP17, SP18, SP19, SP20, SP21. Right panel: Cluster 2 (High-Neuromuscular Male Phenotype, n=7, orange polygon)—athletes SP01, SP02, SP04, SP05, SP06, SP07, SP11. Each panel includes descriptive statistics: age, body weight, height, *n° reps FEMD_INC_, Fpeak FEMD_INC-G_*, Work FEMD_INC-G_*, Dis C7 non-paddle side*. Polygon overlap between Clusters 0 and 2 reflects phenotypic equivalence in neuromuscular dimensions.

Cluster 0 (Moderate-Neuromuscular Male Phenotype, n=4, 19.0%) exhibited balanced profiles across most dimensions, with the athlete roster comprising SP03, SP08, SP09, SP12. Representative phenotypic characteristics included: body weight 79.12 kg, height 179.55 cm, age 21.5 years, *n° reps FEMD_INC_* 13.75, *Fpeak FEMD_INC-G_* 56.70 N, *Work FEMD_INC-G_*8510.34 J, and *Dis C7 non-paddle side* 93.75 cm. This cluster demonstrated moderate-to-high neuromuscular capacity comparable to Cluster 2 in repetitions to failure and total mechanical work, suggesting phenotypic equivalence in sport-specific endurance despite distinct cluster assignments. Note: Athlete SP12, misclassified to Cluster 1 in the held-out test partition (Section 3.2.6), is plotted under its true K-Means assignment.

Cluster 1 (Female-Dominant Phenotype, n=10, 47.6%) demonstrated the smallest radial polygon area, with consistently attenuated normalized values across neuromuscular dimensions. The roster comprised SP10, SP13, SP14, SP15, SP16, SP17, SP18, SP19, SP20, SP21, with representative statistics: body weight 62.48 kg, height 163.30 cm, age 17.9 years, *n°reps FEMD_INC_* 8.20, *Fpeak FEMD_INC-G_* 34.63 N, *Work FEMD_INC-G_ 3062.34* J, and *Dis C7 non-paddle side* 82.95* cm*. This cluster exhibited lower absolute neuromuscular output (40% reduction in peak force, ≈63% reduction in total work relative to Clusters 0 and 2) and reduced anthropometric parameters (body weight 62.48 kg, height 163.30 cm), consistent with normative female physiology (9/10 athletes female) and, secondarily, with the lower mean training age of this group (17.9 years vs. 20.5–21.5 years).

Cluster 2 (High-Neuromuscular Male Phenotype, n=7, 33.3%) displayed maximal polygon expansion across all dimensions, with the roster including SP01, SP02, SP04, SP05, SP06, SP07, SP11. Representative statistics: body weight 77.30 kg, height 176.50 cm, age 20.57 years, *n°reps FEMD_INC_* 13.71, *Fpeak FEMD_INC-G_* 59.17 N, *Work FEMD_INC-G_*8358.19 J, and *Dis C7 non-paddle side* 95.36* cm*. Notably, Cluster 2 demonstrated phenotypic convergence with Cluster 0 across neuromuscular metrics (*Fpeak*: 59.17 vs. 56.70 N; *n° reps*: 13.71 vs. 13.75), reinforcing earlier observations of functional equivalence between these groups despite algorithmic separation.

Comparative analysis across radial profiles is consistent with the tripartite stratification pattern: Cluster 1 occupies a markedly contracted phenotypic space reflecting normative sexual dimorphism and, secondarily, younger training age, while Clusters 0 and 2 exhibit overlapping polygons with near-identical neuromuscular signatures, distinguished exclusively through the secondary DT discriminant (*LD Ts non-paddle side*; Section 3.2.6), a variable absent from the radar visualization; the polygon overlap between Clusters 0 and 2 in **Figure 8** is therefore the expected visual representation of their documented statistical equivalence across all radar-visualized dimensions (all C0–C2: p > 0.05, Section 3.2.5).

#### 3.2.8 Phenotypic cluster interpretation

Based on integrated statistical, discriminant, and visual analyses, three distinct phenotypic profiles emerged with differential implications for talent development and training individualization. Schematic anthropometric archetypes for each cluster are presented in **Figure 9**.

**Figure 9:**
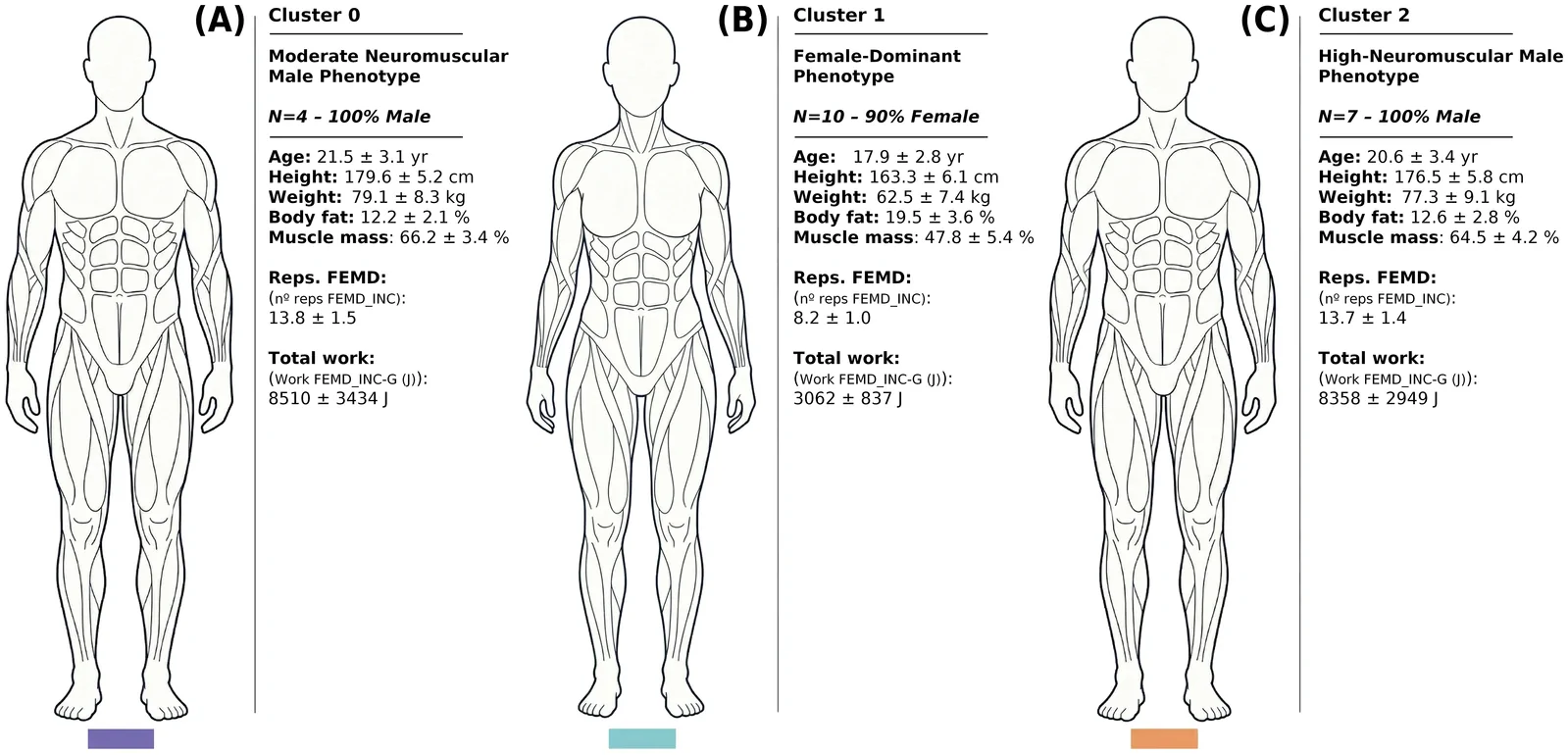
Schematic phenotypic archetypes derived from cluster-mean anthropometric and neuromuscular values (**Table 3** for neuromuscular variables; lean mass estimates from body composition analysis, Section 3.2.3). Silhouettes are standardised anatomical diagrams (frontal view); proportions reflect cluster-mean height, body weight, and lean mass percentage. No facial features are rendered. **(A)** Cluster 0 — Moderate-Neuromuscular Male Phenotype (n=4; 79.1 kg, 179.6 cm, 66.2% lean mass, 13.8 reps, 8510 J). **(B)** Cluster 1 — Female-Dominant Phenotype (n=10; 62.5 kg, 163.3 cm, 47.8% lean mass, 8.2 reps, 3062 J); differences relative to male clusters primarily reflect sexual dimorphism and younger training age (17.9 yr). **(C)** Cluster 2 — High-Neuromuscular Male Phenotype (n=7; 77.3 kg, 176.5 cm, 64.5% lean mass, 13.7 reps, 8358 J). Illustrations are schematic representations only; not photographs of study participants.

Cluster 2 (High-Neuromuscular Male Phenotype, n=7, 33.3%) represents the high-performance archetype characterized by superior neuromuscular capacity (*Fpeak FEMD_INC-G_*: 59.17 N, 71% above Cluster 1), elevated work capacity (*Work FEMD_INC-G_*: 8358 J, 173% above Cluster 1), and greater muscle-contraction sustainment time (LD Ts non-paddle side: 118.2 ms, the lowest value across clusters, indicating more explosive eccentric deceleration capacity). The secondary discriminant separating Cluster 2 from Cluster 0 is *LD Ts non-paddle* side (threshold: ≤152.05 ms; Section 3.2.6; ${\text{p}}_{\text{adj}}$ = 0.105, retained based on RF discriminant importance) (**Figure 9c**). The convergence of mature training age (mean 20.57 years) and developed body mass (77.30 kg) suggests full physiological maturation, positioning this cluster as the competitive performance benchmark.

Cluster 1 (Female-Dominant Phenotype, n=10, 47.6%) exhibits lower absolute neuromuscular output across all FEMD-derived parameters (**Figure 9b**). Given that 90% of this cluster are female athletes, these differences are primarily attributable to normative sexual dimorphism in lean mass, hormonal milieu, and force production capacity, with a secondary contribution from the lower mean training age (17.9 years). Training recommendations for this phenotype must account for sex-specific physiology; neuromuscular development protocols should be calibrated against female-specific normative ranges rather than applied from male athlete benchmarks.

Cluster 0 (Moderate-Neuromuscular Male Phenotype, n=4, 19.0%) occupies a phenotypically equivalent position to Cluster 2 across neuromuscular FEMD dimensions (*n° reps*: 13.75 vs. 13.71; *Fpeak*: 56.70 vs. 59.17 N), with algorithmic separation potentially driven by latissimus dorsi sustain time thresholds (>152.05 ms), unmeasured latent variables (technical efficiency, metabolic economy) or stochastic variance in small-sample clustering (**Figure 9a**). The substantial within-cluster heterogeneity and numerical under-representation (n=4) warrant cautious interpretation, suggesting this cluster may represent algorithmic artifact rather than stable phenotypic subtype. Longitudinal validation with expanded sample sizes would clarify whether Cluster 0 constitutes a genuine intermediate profile or should be consolidated with Cluster 2 under a unified High-Neuromuscular Male Phenotype.

## 4 Discussion

### 4.1 Principal findings and methodological contribution

This investigation identified a tripartite phenotypic structure among international-level flatwater canoeists (n=21) encoding two superimposed sources of biological variation: sexual dimorphism, with the Female-Dominant Phenotype comprising 90% female athletes (χ²=18.90, p<0.001), and intra-sex neuromuscular differentiation between the two exclusively male clusters. The magnitude of this association was further quantified using Cramér’s V, yielding V = 0.949, a value indicating a very strong effect size by conventional benchmarks (Cohen, 1988), which reinforces the characterization of the sex-cluster relationship as a structural confound rather than a marginal statistical association. Internal validation metrics indicated limited cluster separation (silhouette=0.056, well below the commonly cited threshold of ≥0.50 for well-defined clusters (Kaufman and Rousseeuw, 2008), and moderate algorithmic concordance between K-Means and Ward linkage (ARI = 0.591), suggesting that the three-cluster solution, while statistically the most defensible partition, describes overlapping phenotypic gradients rather than discrete categorical profiles. This interpretive constraint should be borne in mind when translating cluster assignments into applied classification frameworks. Decision Tree classification maintained 71.4% test accuracy (5/7 correct; 95% CI: 29.0%–96.3%) with perfect training performance (100%, 14/14; 95% CI: 76.8%–100.0%). While this point estimate significantly exceeds chance-level performance (33.3%; binomial test p=0.045), the wide confidence interval is a direct consequence of the seven-case test partition and constitutes a fundamental statistical limitation of the current validation design. Under a strict frequentist interpretation, the data are consistent with true generalization accuracies ranging from approximately 29% (below chance) to 96%, rendering strong claims of prospective applicability statistically unjustifiable. This uncertainty reflects expected variance in small-sample validation rather than systematic overfitting and reinforces the need for external replication with independent cohorts; LOOCV within the current n=21 sample would not resolve the fundamental instability of small-sample estimation. To illustrate the magnitude of validation data required to meaningfully narrow this interval, halving the observed confidence-interval width while holding the point estimate constant would require approximately a fourfold increase in test-set size (n≈28 versus the current n=7), underscoring that the present validation, while informative as a preliminary indicator, cannot substitute for adequately powered external testing. The primary methodological contribution resides in the deliberate exclusion of all performance variables (_500m_, ranking) from clustering, ensuring purely phenotypic segmentation independent of outcome contamination. The robustness of the statistical characterization was further confirmed by Benjamini-Hochberg False Discovery Rate correction applied across the full 281-variable test battery: 14 of the 15 RF-selected variables retained FDR-corrected significance ( $p_{\text{adj}}$ range: 9.49×10^-6^ to 8.00×10^-^³); *LD Ts non-paddle side* was the sole exception ( $p_{\text{adj}}$ = 0.105), retained on the basis of RF discriminant importance and its role as the secondary Decision Tree node (Section 3.2.5). This conservative approach addresses family-wise error rate inflation inherent to simultaneous testing across high-dimensional phenotypic spaces (n<<p; n=21, p=281; variable-to-case ratio ≈13.4:1), confirming that the reported cluster architecture reflects genuine biological signal rather than statistical artifact of multiple comparisons. It should further be noted that Random Forest variable importance rankings, like the inferential tests above, are subject to instability under high-dimensional, small-sample conditions; the reported top-15 ranking should therefore be interpreted as indicative of relative discriminant contribution within this specific sample rather than as a stable, generalizable ordering of phenotypic determinants. This instability was empirically quantified via a bootstrap resampling procedure (B = 1000 resamples of the n=21 sample, with the Random Forest re-fitted and feature importance recomputed at each iteration): on average, only 36.9% (SD = 12.7%; median=40.0%; 95% CI: 13.3%–60.0%) of the originally reported top-15 variables were retained in the top-15 ranking across resamples, providing direct empirical confirmation — rather than a theoretical caveat alone — of the ranking instability expected under the present high-dimensional, small-sample conditions (n=21, p=281). It is also worth noting that the modest absolute sample size (n=21) reflects the inherently narrow global population of internationally competitive flatwater canoeists rather than a recruitment shortfall, a contextual factor elaborated further in Section 4.6.

The three-cluster solution revealed: (1) Cluster 2 — High-Neuromuscular Male Phenotype (n=7, 33.3%): superior neuromuscular capacity (*Fpeak FEMD_INC-G_*: 59.17 N; *Work FEMD_INC-G_*: 8358 J); (2) Cluster 1 — Female-Dominant Phenotype (n=10, 47.6%): lower absolute force output consistent with normative sexual dimorphism (34.63 N; 3062 J); (3) Cluster 0 — Moderate-Neuromuscular Male Phenotype (n=4, 19.0%): phenotypically equivalent to Cluster 2 in neuromuscular metrics (56.70 vs. 59.17 N; 13.75 vs. 13.71 reps) despite algorithmic separation. The top 15 discriminant variables explained 39.4% of cumulative Random Forest importance; the domain-level breakdown of top-15 importance yielded: neuromuscular 75.9%, trunk flexibility 7.1%, metabolic-anthropometric 6.3%, body composition 5.8%, and muscle contractile properties 4.8%.

### 4.2 Neuromuscular determinants and functional interpretation

FEMD-derived variables dominated cluster discrimination (75.9% of top-15 cumulative importance), with *Fpeak FEMD_INC-G_* exhibiting the highest discriminant capacity (importance=0.061), followed by *n° reps FEMD_INC_* (0.033) and *Work FEMD_INC-G_* (0.032), collectively explaining 12.6% of total importance. These findings align with biomechanical models wherein stroke-specific power output and fatigue resistance are primary determinants of water velocity in sprint canoeing (Álvarez-Yates et al., 2026; Álvarez-Yates et al., 2024b). Differences in absolute FEMD output between the Female-Dominant Phenotype (Cluster 1) and the two male phenotypes (Clusters 0 and 2) are substantial but must be interpreted within the context of sexual dimorphism. *Fpeak FEMD_INC-G_* differed markedly (34.6 ± 3.2 N vs. 56.7 ± 10.6 N and 59.2 ± 8.5 N; p<0.001), as did mechanical work capacity (3062.3 vs. 8510.3 and 8358.2 J) and repetitions to failure (8.2 vs. 13.8 and 13.7 reps). Body-weight normalization attenuated but did not eliminate these differences (*n° reps FEMD_INC/kg_*: 0.134 vs. 0.182 and 0.177 reps/kg; 27% reduction), consistent with established evidence that sex-dependent variation in muscle fibre cross-sectional area and hormonal milieu cannot be fully corrected by mass scaling alone (Hunter, 2014; Staron et al., 2000). The more informative comparison for performance-related phenotypic differentiation is between the two exclusively male clusters. Clusters 0 and 2 demonstrated complete statistical equivalence across all 15 pairwise comparisons (all p > 0.05; **Table 3**), including mean eccentric power (*Pm FEMD_INC-E_*: 268.2 ± 55.3 vs. 264.5 ± 44.0 W; p = 0.988), with no variable reaching significance between these phenotypes. This near equivalence suggests that the algorithmic separation of Clusters 0 and 2 reflects subtle multivariate differences, potentially in trunk stability efficiency or metabolic economy, rather than a meaningful neuromuscular performance gradient within the male cohort.

### 4.3 Body composition and anthropometric contributions

The body composition profile of Cluster 1, elevated adiposity (19.5 ± 3.6%), reduced lean mass (47.8 ± 5.4%), and lower absolute *FEMD_ISO_* (1932.7 ± 321.5 N·s vs. 2664.6 ± 216.2 and 3023.0 ± 438.8 N·s; noting that the Cluster 0–Cluster 1 difference did not reach statistical significance, p = 0.067; only the Cluster 1–Cluster 2 contrast was significant, p = 0.001), is consistent with normative female physiology (Hunter, 2014; Staron et al., 2000) rather than indicative of athletic underperformance. Female athletes characteristically exhibit 10–13% essential fat stores vs. 2–5% in males, and approximately 40–60% lower absolute upper-body force production capacity, patterns that align precisely with the Cluster 1 profile. Body-mass normalization attenuates absolute differences but cannot fully correct for sex-dependent variation in muscle fibre cross-sectional area, androgenic hormonal milieu, or biomechanical lever arm geometry. Accordingly, the 38% lower muscle-to-fat ratio and the attenuated body-weight-normalized dynamic strength (*1RM/kg bench press*: 1.2 ± 0.1 vs. 1.6 ± 0.2 and 1.5 ± 0.2, (Section 3.2.3)) observed in Cluster 1 should be interpreted as reflections of sexual dimorphism rather than as modifiable performance deficits. Body composition differentiation was statistically profound across all metrics (all p<0.001): muscle percentage 47.8 ± 5.4% vs. 66.2 ± 5.8% (Cluster 0) and 64.5 ± 5.8% (Cluster 2); body fat 19.5 ± 3.6% vs. 12.2 ± 2.3% and 12.6 ± 1.9% (Section 3.2.3). Distance from C7 to fingertips displayed an inverse association with cluster membership: Cluster 1 demonstrated significantly reduced arm length (83.0 ± 3.8 cm vs. 93.8 ± 2.8 and 95.4 ± 1.9 cm, p<0.001). Muscle contractile properties (*LD Ts non-paddle side*) revealed a contrasting pattern: Cluster 1 exhibited greater muscle sustain time in LD relative to Cluster 2 (196.1 ± 81.2 vs. 118.2 ± 27.3; Dunn p = 0.023), suggesting elevated LD Ts in the female-dominant phenotype (indicative of slower contractile properties) and lower LD Ts in the high-neuromuscular male phenotype (indicative of faster, more explosive contractile capacity). This difference did not survive FDR correction ( $p_{\text{adj}}$ = 0.105), and the high within-group variability in Cluster 1 (SD = 81.2) limits inferential strength.

The tripartite cluster structure therefore encodes two biologically meaningful axes of variation: sexual dimorphism (Cluster 1 vs. Clusters 0 and 2) and intra-male neuromuscular differentiation (Cluster 0 vs. Cluster 2), both phenotypically valid and informative for athlete profiling. Future studies should explicitly stratify cluster analyses by sex, or incorporate sex as a covariate in ANCOVA frameworks, to isolate developmental maturity effects from constitutional phenotypic differences.

### 4.4 Cluster stability and classification interpretability

The ARI of 0.591 between K-Means and hierarchical Ward linkage indicates moderate agreement beyond chance but falls short of the ≥0.80 threshold conventionally considered indicative of strong concordance (Vinh et al., 2010). This moderate concordance, combined with the low silhouette coefficient (0.056), implies that the three-cluster boundary solution is sensitive to algorithmic choice and initialization parameters, with a non-trivial proportion of individual assignments differing between methods. Consequently, individual athlete cluster membership should be interpreted as a probabilistic tendency rather than a deterministic categorical assignment, particularly for athletes whose feature vectors fall proximal to inter-cluster decision boundaries (e.g., SP12, SP04).

Decision Tree analysis (*max_depth=3*) achieved 100% training accuracy (14/14) and 71.4% test accuracy (5/7), with 90.5% overall correct classification (19/21 athletes). The interpretable two-threshold architecture encodes candidate classification rules: (1) root node split on *Fpeak FEMD_INC-E_* ≤39.48 N segregates the Female-Dominant Phenotype (Cluster 1, 42.9% of the training partition); (2) secondary bifurcation on *LD Ts non-paddle side* refines classification between the High-Neuromuscular Male Phenotype (Cluster 2) and the Moderate-Neuromuscular Male Phenotype (Cluster 0) within the exclusively male subspace.

The identification of *Fpeak FEMD_INC-E_* as the root discriminant offers biomechanical insight into canoeing-specific neuromuscular demands. The threshold ≤39.48 N is, however, calibrated on a mixed-sex sample and primarily encodes sexual dimorphism in absolute force production: female athletes normatively produce lower eccentric force regardless of competitive level. Within the male phenotypic subspace, eccentric capacity appears to function as a prerequisite for effective force transmission: in the unstable C1 kneeling position, the ability to eccentrically decelerate the trunk during the recovery phase is mechanistically linked to lumbopelvic stability under dynamic loading. Athletes near or below this threshold within a sex-stratified male sample may exhibit reduced capacity for trunk stabilization during high-load phases, potentially limiting effective concentric force expression. Sex-specific eccentric force thresholds should be established before applying this classification rule prospectively to female athletes. Misclassifications involved SP12 (Cluster 0→1) and SP04 (Cluster 2→0), both exhibiting feature values proximal to decision thresholds, consistent with the overlapping phenotypic gradients described in Section 4.1. All Cluster 1 athletes in the test partition were correctly classified, reflecting the magnitude of sex-normative differences in absolute force production rather than discrete categorical separation. These classification instabilities are also consistent with the constrained phenotypic diversity that characterizes elite performance populations, where restriction of range relative to the general population inherently compresses inter-individual variance and may attenuate cluster separability independently of sample size (Mesquida et al., 2022).

### 4.5 Practical applications and future directions

The parsimonious two-threshold classification algorithm offers a candidate framework for prospective athlete phenotyping via standard laboratory equipment (FEMD, dynamometer) without computational infrastructure, pending external validation, potentially informing:

1. Sex-stratified talent identification: rapid classification into established phenotypic profiles should be conducted within sex-specific reference frameworks. The eccentric force threshold (39.48 N) is calibrated on a mixed-sex sample and requires sex-stratified recalibration before prospective application to female athletes.
2. Training individualization: within-sex phenotypic classification should guide programming. Female-dominant phenotype athletes (Cluster 1) require neuromuscular development benchmarks calibrated against female-specific normative ranges, with eccentric capacity development as a priority within sex-appropriate loading parameters — not as an attempt to reach male-derived thresholds. Cluster 0 males may benefit from trunk stability optimization to reduce lateral displacement (based on the secondary decision tree discriminant *LD Ts non-paddle side*; pending FDR-significant replication, Section 3.2.5); Cluster 2 males represent the current high-neuromuscular benchmark within the male cohort.
3. Longitudinal monitoring: repeated classification across macrocycles can detect phenotypic transitions, particularly within the male subspace where intra-sex phenotypic differentiation between Clusters 0 and 2 provides the most sensitive monitoring space for detecting developmental transitions.

Radar chart visualization (**Figure 8**) provides an intuitive communication tool for multidisciplinary sport science teams, integrating normalized profiles (0.1–0.9 scale), athlete rosters, and cluster-specific descriptive statistics into a single accessible display.

Future research should prioritize: (a) sex-stratified replication of the cluster architecture with adequately powered, sex-balanced cohorts to isolate training-related phenotypic variation from normative biological dimorphism; (b) randomized trials assigning cluster-specific interventions to empirically test phenotype-tailored programming superiority versus standardized approaches; (c) longitudinal tracking of cluster transitions to establish developmental trajectories within each sex.

### 4.6 Limitations and methodological considerations

The internal cluster validity metrics — silhouette=0.056 and Davies-Bouldin=2.265 — indicate substantial intra-cluster heterogeneity and limited inter-cluster separation. Silhouette values at or below 0.25 are generally interpreted as evidence that no substantial cluster structure has been found (Kaufman and Rousseeuw, 2008); the present value of 0.056 falls substantially below this threshold, placing the solution within the range of absent or indeterminate structure by conventional criteria. While analogously low silhouette values have been documented in elite sport clustering studies (Mausehund et al., 2025), this observation does not constitute validation of the solution but rather reflects the inherent phenotypic continuum of high-performance populations. The three-cluster solution should therefore be understood as a data-reduction heuristic identifying the most statistically defensible segmentation of a continuous phenotypic space, rather than as evidence of discrete, biologically meaningful athlete categories. External validation via longitudinal follow-up or independent cohort replication is required before cluster assignments can support consequential decisions in talent identification or training individualization.

The most substantive limitation of this study is the structural confounding of sex with cluster assignment: Cluster 1 comprises 90% female athletes while Clusters 0 and 2 are exclusively male. Although this sex-cluster association is explicitly incorporated into the interpretive framework, with the tripartite structure reconceptualised as encoding sexual dimorphism alongside intra-sex neuromuscular differentiation between male phenotypic subgroups, the current analytical design cannot quantify the relative contributions of normative biological dimorphism versus sport-specific training adaptations to the observed inter-cluster differences. Body-mass normalization was applied to neuromuscular variables but cannot fully correct for sex-dependent variation in muscle fibre composition, androgenic hormonal milieu, or biomechanical lever geometry. Future research must employ sex-stratified cluster analyses with adequately powered, sex-balanced cohorts to isolate training-related phenotypic variation from constitutional biological differences. A related, partially independent source of confounding arises from the wide age range within the analytic sample (15–36 years), which spans markedly distinct developmental and training-maturation stages. Because Cluster 1 (Female-Dominant Phenotype) also exhibits a lower mean training age (17.9 years) relative to Clusters 0 and 2 (21.5–20.5 years; Section 3.2.7), chronological and training-age maturation covaries with both sex and cluster assignment, precluding independent attribution of the observed neuromuscular and anthropometric differences to sex, developmental maturity, or their interaction. Future sex-stratified replications should additionally stratify or statistically control for training age (e.g., via ANCOVA with age as a covariate) to disentangle these superimposed sources of biological variance.

Modest sample size (n=21) with a small test partition (n=7) introduces substantial variance in accuracy estimates: each misclassification represents a 14.3-percentage-point swing, directly producing the wide Clopper-Pearson confidence interval (71.4%; 95% CI: 29.0%–96.3%). Although accuracy significantly exceeded chance-level performance (p_0_=1/3; p=0.045), the confidence interval encompasses values both below and substantially above chance, underscoring that the point estimate should not be interpreted as a stable measure of generalization performance. External replication with independent cohorts is required; LOOCV within the current n=21 sample would yield per-fold test partitions of n=1 and would not resolve the fundamental instability of small-sample accuracy estimation. This constraint should be contextualized against the inherently narrow population of internationally competitive flatwater canoeists relative to general-population research, and against the fact that median sample sizes reported in elite sports-science and biomechanics research are comparably constrained (median n=19 in the Journal of Sports Sciences; mean sample sizes ranging 15–32 across four additional biomechanics and sports-science journals surveying 188 studies) (Mesquida et al., 2022) — though it should be acknowledged that comparably small sample sizes have themselves been identified as a contributing factor to replicability concerns in the field, reinforcing rather than mitigating the need for the external validation emphasized throughout this Discussion.

Additional limitations include: (a) cross-sectional design precluding causal inference regarding whether phenotypic differences drive performance or emerge from differential training histories; (b) omission of psychological attributes (competitive mindset, pain tolerance), technical proficiency (stroke efficiency), and tactical acumen (pacing strategy), limiting deterministic performance prediction from phenotypic cluster membership alone; (c) Cluster 0’s numerical under-representation (n=4) and within-cluster heterogeneity, which may reflect algorithmic partition rather than a stable phenotypic subtype; longitudinal validation with expanded sample sizes is required to determine whether Cluster 0 constitutes a genuine intermediate profile or should be consolidated with Cluster 2.

### 4.7 Conclusion

Unsupervised machine learning applied to comprehensive laboratory phenotyping of elite flatwater canoeists suggests a tripartite phenotypic structure that appears to encode two biologically meaningful axes of variation: sexual dimorphism, separating the Female-Dominant Phenotype (Cluster 1, 90% female) from the two exclusively male clusters; and intra-sex neuromuscular differentiation between the High-Neuromuscular Male Phenotype (Cluster 2) and Moderate-Neuromuscular Male Phenotype (Cluster 0). FEMD-derived neuromuscular variables dominated cluster discrimination (75.9% of top-15 cumulative importance), with peak eccentric force as the primary classification node, encoding both sex-normative force differences and sport-specific neuromuscular capacity. The three-cluster solution exhibits moderate algorithmic concordance (ARI = 0.591) and interpretable classification rules (71.4% test accuracy; 95% CI: 29.0%–96.3%), with the wide confidence interval reflecting the inherent statistical limitations of seven-case validation rather than systematic model failure. By excluding all performance variables from clustering, this study shows that phenotypic differences among elite canoeists can be detected independently of competitive outcomes, within the constraints of this exploratory sample. The Moderate-Neuromuscular Male Phenotype (Cluster 0, n=4) requires longitudinal validation to determine whether it constitutes a stable phenotypic subtype or an algorithmic partition of the continuous male neuromuscular space. Future research should replicate the cluster architecture within adequately powered, sex-balanced cohorts to isolate training-related phenotypic variation from normative biological dimorphism and employ independent external validation to establish the prospective generalizability of the proposed classification rules.

## Data Availability

The raw data supporting the conclusions of this article will be made available by the authors, without undue reservation.
