# Supplementary material for: Unsupervised phenotypic clustering, inferential characterization, and discriminant classification of elite flatwater canoeists: neuromuscular and anthropometric determinants
Source: Front Physiol. 2026 Oct 19;17:1949031. doi: 10.3389/fphys.2026.1949031 (PMC13725060; doi:10.3389/fphys.2026.1949031)
Supplement: Supplementary file 1 [file Table1.docx]

Unsupervised Phenotypic Clustering, Inferential Characterization, and Discriminant Classification of Elite Flatwater Canoeists: Neuromuscular and Anthropometric Determinants

Tania Álvarez-Yates ^1,2^, Óscar García-García ^2,3*^, Alba Cuba-Dorado ^2,3^, José-Benito Bouza-Rodríguez ^4^, Alberto Comesaña-Campos ^4^ and Jorge Cerqueiro-Pequeño ^4^

^1^ Department of Physical Education and Sports, Faculty of Sports Sciences and Physical Education, University of A Coruna, A Coruna, Spain.

^2^ Sports Performance, Physical Condition and Wellness lab, Faculty of Education and Sport Sciences, University of Vigo, Pontevedra, Spain.

^3^ Sports Research Institute (CIDEGA), Faculty of Education and Sport Sciences, University of Vigo, Pontevedra, Spain.

^3^ Department of Design in Engineering, School of Industrial Engineering, University of Vigo, Vigo, Spain.

*** Correspondence:**

Óscar García-García
oscargarcia@uvigo.gal

# 1 SUPPLEMENTARY MATERIAL

**Supplementary Table 1** summarizes the operational meaning of prefixes and suffixes describing trunk stability, scapular control, pelvic and femoral control, ankle–foot contribution, global center-of-mass stability, and flexibility/mobility tests (Y-Balance, shoulder ROM, trunk flexion, Active Knee Extension)

**Supplementary Table 2** details the nomenclature of neuromuscular variables from FEMD traction and press tests (concentric, eccentric, and global phases), including load, force, velocity, power, work, impulse, time under tension, and body-mass-normalized indices derived from isometric and incremental canoe-specific assessments.

**Supplementary Table 1.** Operational description of TMG and mobility variables

| Prefix / suffix | Operational meaning (summary) |
| --- | --- |
| BF* | Biceps femoris |
| DE* | Deltoid |
| ES* | Erector spinae |
| LD* | Latissimus dorsi |
| PM* | Pectoralis mayors |
| RF* | Rectus femoris |
| ST* | Semitendinosus |
| TA* | Tibial anterior |
| TZ* | Trapezius |
| Paddle | Measurement on the paddle side (canoe paddling dominant side). |
| Non-paddle | Measurement on the non‑paddle side (contralateral side). |
| ASIM | Asymmetry indices between paddle and non‑paddle side. |
| SIM | Global symmetry indices or composite symmetry scores. |
| RE* | Shoulder external rotation, reported for paddle side, non‑paddle side and/or asymmetry indices. |
| RI* | Shoulder internal rotation, analogously reported by side and asymmetry. |
| FLX* | Shoulder flexion range of motion and control, by side and asymmetry indices. |
| Dist.C7* | Distance from C7 spinous to fingertip reported for paddle side arm and non‑paddle side. |
| YBT* | Y‑Balance Test variables (anterior, posteromedial, and posterolateral reach; paddle side, non‑paddle side, and asymmetries). |
| AKE* | Active Knee Extension test: active knee extension range in supine position, by side and asymmetry. |

**Supplementary Table 2.** Operational description of explosive strength and FEMD‑derived variables

| Prefix / suffix | Operational meaning (summary) |
| --- | --- |
| Kg cable-row | External load used in the single arm cable row (kg). |
| Vm cable-row* | Mean velocity in single arm cable row |
| Vmax cable-row* | Peak velocity achieved during single arm cable row. |
| Pm cable-row* | Mean power output during single arm cable row. |
| Pmax Trac.* | Peak power during single arm cable row. |
| Fm cable.row* | Mean force during the single arm cable row. |
| Fmax cable.row* | Peak force single arm cable row. |
| Kg leg-press | External load used in the leg press (multi‑joint pushing exercise). |
| Vm leg-press* | Mean velocity during the leg press. |
| Vmax leg-press* | Peak velocity during the leg press. |
| Pm leg-press* | Mean and peak power during the press. |
| Pmax leg-press* | Peak power achieved in leg press. |
| Fm leg-press* | Mean force during the leg press. |
| Fmax leg-press* | Peak force in leg press. |
| FEMD_ISO_ | Canoe-specific isometric functional electromechanical dynamometer test. |
| Fm FEMD_ISO_ | Mean isometric force in canoe‑specific position. |
| Fpeak FEMD_ISO_ | Peak isometric force in canoe‑specific position. |
| Tpeak FEMD_ISO_ | Time to peak force in the isometric test. |
| Imp FEMD_ISO_ | Impulse (force–time integral) in the isometric test. |
| FEMD_ISO/Kg_ | Body‑mass‑normalized versions of the isometric variables. |
| FEMD_INC_ | Canoe-specific incremental functional electromechanical dynamometer test. |
| nº reps FEMD_INC_ | Number of repetitions in the canoe‑specific incremental traction test to failure. |
| nº reps FEMD_INC/Kg_ | Number of repetitions in the canoe-specific incremental traction test to failure, normalized to body mass (rep·kg⁻¹). |
| Tens FEMD_INC-c_ | Total time under tension in the concentric phase of the incremental test. |
| Fm FEMD_INC-c_ | Mean force in the concentric phase of the incremental test. |
| Fpeak FEMD_INC-c_ | Peak force in the concentric phase of the incremental test. |
| Vm FEMD_INC-c_ | Mean velocity in the concentric phase. |
| Vpeak FEMD_INC-c_ | Peak velocity in the concentric phase. |
| Pm FEMD_INC-c_ | Mean power in the concentric phase. |
| Ppeak FEMD_INC-c_ | Peak power in the concentric phase. |
| Work FEMD_INC-c_ | Total mechanical work in the concentric phase. |
| Imp FEMD_INC-c_ | Impulse (force–time integral) in the concentric phase. |
| FEMD_INC-c/kg_ | Body‑mass‑normalized concentric incremental variables. |
| Tens FEMD_INC-E_ | Time under tension in the eccentric phase. |
| Fm FEMD_INC-E_ | Mean force in the eccentric phase. |
| Fpeak FEMD_INC-E_ | Peak force in the eccentric phase. |
| Pm FEMD_INC-E_ | Mean power in the eccentric phase. |
| Ppeak FEMD_INC-E_ | Peak power in the eccentric phase. |
| Work FEMD_INC-E_ | Work in the eccentric phase. |
| Imp FEMD_INC-E_ | Impulse in the eccentric phase. |
| FEMD_INC-E/Kg_ | Body‑mass‑normalized eccentric incremental variables. |
| Tens FEMD_INC-G_ | Total time under tension (concentric + eccentric). |
| Fm FEMD_INC-G_ | Mean global force in the incremental test. |
| Fpeak FEMD_INC-G_ | Peak global force in the incremental test. |
| Vm FEMD_INC-G_ | Mean global velocity. |
| Vpeak FEMD_INC-G_ | Peak global velocity. |
| Pm FEMD_INC-G_ | Mean global power. |
| Ppeak FEMD_INC-G_ | Peak global power. |
| Work FEMD_INC-G_ | Total work and impulse over the entire incremental test. |
| Imp FEMD_INC-G_ | Impulse over the entire incremental test. |
| FEMD_INC-G/Kg_ | Body‑mass‑normalized global incremental variables. |
